# Sequence-controlled divergent supramolecular assembly of polyproline helices into metallo-peptide nanoparticles†

**DOI:** 10.1039/d4na00762j

**Published:** 2024-12-05

**Authors:** Dominic F. Brightwell, Kushal Samanta, Julie A. Watts, Michael W. Fay, Aniello Palma

**Affiliations:** a School of Chemistry, University College Dublin Belfield Dublin 4 Ireland aniello.palma@ucd.ie; b School of Chemistry and Forensic Science, Supramolecular and Interfacial Chemistry, Ingram Building, The University of Kent Canterbury CT2 7NZ Kent UK; c School of Chemistry, University of Birmingham Edgbaston Birmingham B15 2TT UK; d Nanoscale and Microscale Research Centre, University of Nottingham Nottingham NG7 2RD UK; e School of Pharmacy, University of Nottingham Nottingham NG7 2RD UK; f Faculty of Engineering, University of Nottingham Nottingham NG7 2RD UK

## Abstract

The field of peptide based supramolecular biomaterials is fast evolving. These types of constructs have been shown to find applications in the fields of bioimaging, drug delivery and scaffolds for chemical reactions. However, the community typically focuses on the use of two specific classes of structured peptides: α-helices and β-sheets, clearly neglecting a unique peptide secondary structure: the polyproline helix. Herein, we report the first design, synthesis and characterization of polyproline based metallo-peptide nanoparticles. We demonstrate that rationally engineered polyproline helices can assemble in a divergent manner, into two types of nanoparticles. We also demonstrate that the primary sequence of the functionalised polyproline peptide is crucial to ensure a controlled assembly. This work clearly demonstrates that polyproline helices can be a powerful tool to achieve supramolecular assemblies of complex and responsive bioinspired nanomaterials.

## Abstract 2

We report the divergent assembly of polyproline helices into two types of metallo-peptide nanoparticles. In this report we also show that the primary sequence is crucial to ensure a controlled and desired assembly.

## Graphical abstract

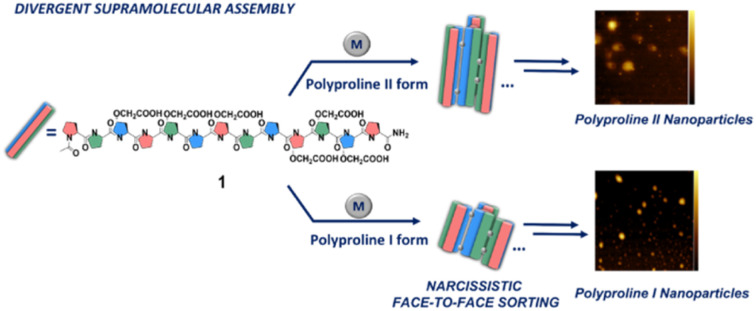

## Introduction

**Fig. 1:**
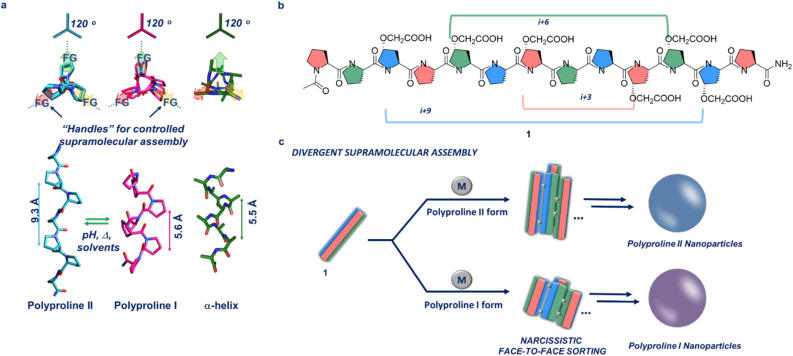
(a) Side and top view of a polyproline II, left-handed helix with all trans conformations of the amide bonds and polyproline I, right-handed helix with all cis conformations of the amide bonds. Comparison with an α-helix, a secondary structure commonly used in supramolecular chemistry; (b) polyproline 1 with the three faces of the helix colour coded. On each face there are two chemical handles (*i.e.* carboxylic acids) spaced at *i* + 3; *i* + 6 and *i* + 9 respectively; (c) Divergent Supramolecular Assembly: schematic representation of the metal driven divergent supramolecular assembly of 1 in the polyproline I and II forms into nanoparticles *via* a narcissistic metal-driven and face-selective approach.

The field of supramolecular bioinspired materials has grown immensely in recent years. Biomolecules such as peptides, proteins, lipids and DNA/RNA, have been used as supramolecular building blocks in the synthesis of novel supramolecular biomaterials.^1–6^ Within this area of research, a considerable amount of effort has been dedicated towards creating reliable, rational methods for designing hierarchical supramolecular constructs using structured peptides (*i.e.* peptides with defined secondary structures).^4,7,8^ These peptide-based supramolecular assemblies have found application in the field of bioimaging, drug delivery and scaffolds for chemical reactions.^9–11^ As the field developed, efforts have been made to move from an empirical and observation design to a rational and predictive one.^12^ The works of Nowick on β-sheet self-assembly, Woolfson on α-helix bundles and Gazit on super-helical structures are elegant examples of this type of rational design approach.^13–15^ However, while α-helices and β-sheets have been extensively used as supramolecular building blocks, the polyproline helix's secondary structure has been largely overlooked.^12,16^ Polyproline helices are secondary structures which appear in most proteins. They have a similar occurrence in nature to 3_10_ and π-helices. We have recently demonstrated that polyproline helices can be rationally designed and functionalized, allowing predictable tuning of supramolecular interactions, to engineer the formation of supramolecular peptide frameworks for guests encapsulation and chiral separation, as well as Pd_2_L_4_ type cages.^17–19^ Excellent work in the field of supramolecular chemistry using polyproline has been done by the Wennemer's team with examples of polyproline MOF based systems and polyproline based supramolecular catalysts.^20–22^ Unique to this type of peptide, is its ability to interconvert between two different stable conformations as a function of the environment it is exposed to *i.e.* temperature, solvent polarity and pH (Fig. 1a, polyproline II, left-handed helix with all *trans* conformations of the amide bonds and polyproline I, right-handed helix with all *cis* conformations of the amide bonds).^23^ This interconversion occurs *via cis*–*trans* isomerisation of the amide bonds and leads to a change in the chirality (left-*vs.* right-handed helix) and size of the biopolymer (polyproline II is ≈1.6 times longer than polyproline I). Moreover, similarly to α-helices, polyproline I and II have three faces which can be accurately functionalised to induce the desired controllable self-assembly (Fig. 1a). Several reports have appeared in the literature in which the controlled self-assembly or metal-driven assembly of α-helices, have been used to yield 2D and 3D supramolecular constructs.^24–26^ The ability for polyproline helices to interconvert into two stable and distinct secondary structures, combined with the presence of three faces for both polyproline forms, similar to α-helices (Fig. 1a), inspired the idea to exploit this class of peptides as powerful supramolecular building blocks. In this work, we take advantage of their high level of resilience to functionalization, to demonstrate that rationally engineered polyproline helices can assemble in a divergent manner, demonstrating that polyproline helices are powerful and versatile supramolecular building blocks.

## Results and discussion

Our aim is to assemble the polyprolines in a controlled manner *via* a narcissistic metal-driven and face-selective approach (*i.e.* the same face of two different polyproline helices interacting together, Fig. 1c). This face-to-face assembly mode aims to mimic the supramolecular behaviour of engineered α-helices which engage intramolecularly to yield extended 3D nanostructures.^24–26^ To achieve this goal, polyproline 1, with two chemical handles *per* face on its backbone was designed and synthesised. By design, these chemical handles have different spacing on each of the three faces of the polyproline helix, to promote a narcissistic face-to-face self-sorting (Fig. 1b). The chemical handle selected for this study is a carboxylic acid, introduced on the proline *via* facile functionalisation of commercially available *trans*-4-Hydroxyl-l-proline (Scheme SI1†). Addition of appropriate metal ions to 1, induces the formation of a series of metalorganic nodes between polyproline helices, promoting an extended assembly.

**Fig. 2:**
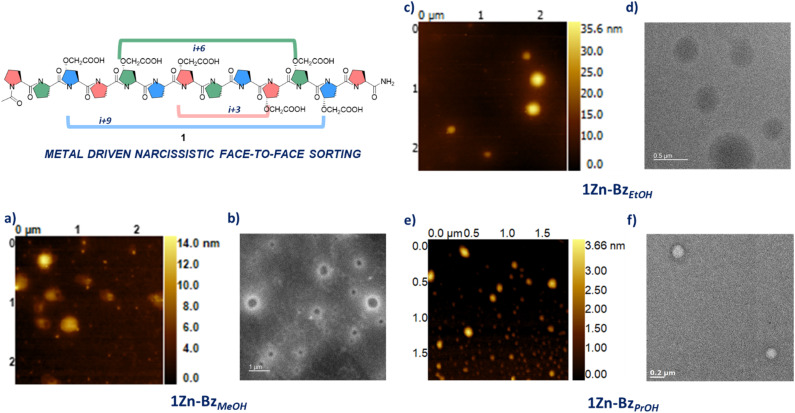
(a) AFM of 1Zn-Bz_MeOH_; (b) DF-STEM of 1Zn-Bz_MeOH_. Sample is not stained; (c) AFM of 1Zn-Bz_EtOH_; (d) TEM of 1Zn-Bz_EtOH_. Sample is not stained; (e) AFM of 1Zn-Bz_PrOH_; (f) TEM of 1Zn-Bz_PrOH_. Sample is not stained.

**Fig. 3:**
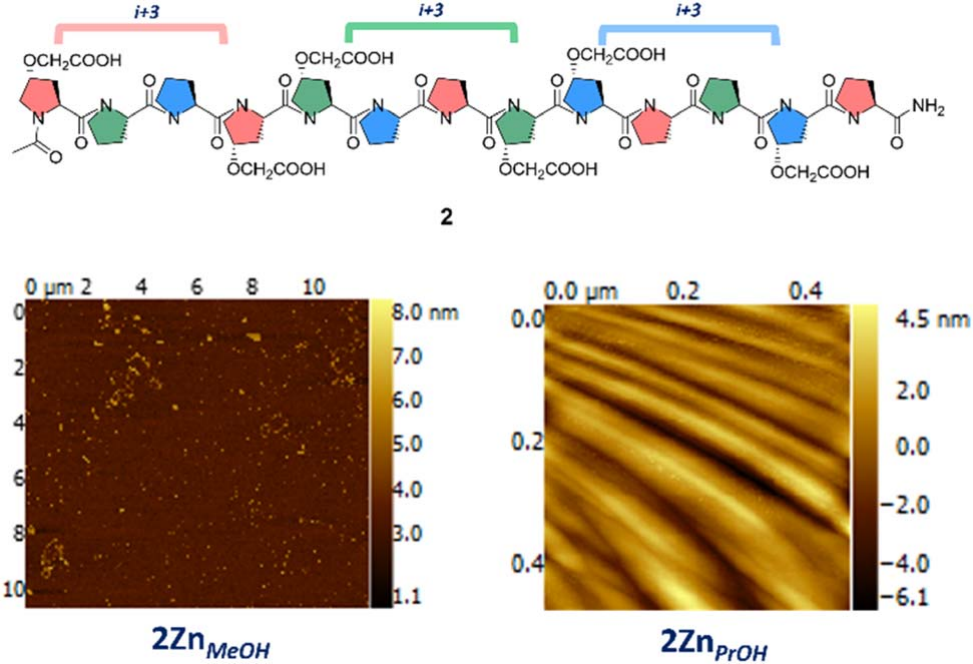
Peptide 2 assembled in methanol and propanol in the same conditions as 1 (*i.e.* triethylamine and zinc nitrate). Bottom left panel: AFM of 2Zn_MeOH_; bottom right panel AFM of 2Zn_PrOH_.

Peptide 1 was synthesised using solid phase peptide synthesis (SPPS) using a Rink amide resin. After cleavage the peptide was purified using reverse phase HPLC. 1 was incubated for fourteen days in water, methanol, ethanol and propanol, and circular dichroism (CD) spectra were recorded. These solvents were selected as we intend to investigate the ability of 1 to switch between polyproline form II, typically favoured by water and methanol, and polyproline form I typically found in aliphatic alcohols such as propanol and in certain cases ethanol.^27–29^ The water, methanol and ethanol solutions showed that 1 was found predominantly in the polyproline II form (min at 206 nm and max at 225 nm for methanol; min at 204 nm and max at 224 nm for ethanol), while the propanol solution showed a minimum at 200 nm and a maximum at 216 nm typical of polyproline I (Fig. SI17†). As the metal driven assembly requires the carboxylate form of the carboxylic acids, triethylamine was selected as a base. CD spectra of 1 incubated in the same solvents with triethylamine, clearly showed the adoption of the polyproline I helix for the propanol solution while the methanol solution showed mainly 1 in the polyproline II form (Fig. SI18†). Interestingly, the triethylamine ethanolic solution gave a CD indicative of a partial conversion of 1 into the polyproline I form (min at 203 nm and max at 220 nm for ethanol, Fig. SI18†). These results are quite remarkable as they highlight the resilience of the polyproline secondary structure, as we were able to insert six non-natural amino acids (*i.e.* 46% of the backbone for 1) without altering its' secondary structures nor the ability of 1 to interconvert between the I and II forms. With these results in hand, we had a defined set of solvents to use in our attempt to perform a divergent supramolecular assembly of 1 into nanoparticles. Stock solutions of 1 in water, methanol, ethanol and propanol, with triethylamine (1 : 1 ratio of carboxylic acids to NEt_3_) were treated with zinc nitrate (6 : 3 ratio of carboxylic acids to zinc ions) at room temperature to trigger the metal driven assembly. The solutions obtained (≈0.4 mg ml^−1^) were initially analysed with dynamic light scattering (DLS). DLS for the water solution 1Zn_aq_ indicated the presence of particles with an average size of 211 nm and an Average Polydispersity Index (PdI) of 0.407 (Fig. SI1†). Subsequent AFM analysis of this sample showed the presence of small nanoparticles as well as regions with large aggregates (Fig. SI18†). Despite the poor polydispersity and the presence of aggregates, we were pleased to see that the polyproline 1 assembled as expected into nanoparticles. To address the polydispersity issue encountered with the aqueous solution, we decided to use methanol as, 1 was also in the desirable polyproline II conformation in this solvent. DLS analysis of 1Zn_MeOH_, showed the presence of nanoparticles with an improved dispersity (Average size 98.2 nm; Average PdI 0.190, Fig. SI2†). To assess how 1 in a mix conformation of polyproline I and II forms, would impact the supramolecular assembly process, the basic ethanol solution of 1 was also treated with zinc nitrate. DLS analysis of 1Zn_EtOH_ showed the presence of nanoparticles with an improved dispersity compared to the aqueous solution (average size 208.7 nm; Average PdI 0.217, Fig. SI4†). The polydispersity of these samples was indicative of defects in the supramolecular network. Treatment of these solutions with a limiting amount of a modulator such as benzoic acid (6 : 1 ratio of carboxylic acid to benzoic acid) improved the dispersity for both samples (Fig. SI3† average size 139.2 nm; average PdI 0.122 for 1Zn-Bz_MeOH_ and Fig. SI5† Average size 208.6 nm; average PdI 0.095 for 1Zn-Bz_EtOH_).^30^ These solutions were analysed using atomic force microscopy (AFM) in tapping mode. 1Zn-Bz_MeOH_ presented nanoparticles with a diameter ranging between 233 nm and 330 nm (Fig. 2a). Maximum height for these particles ranged between 8 nm and 16 nm, indicating these were flattened disks and in some cases buckling was clearly visible (Fig. SI12†), suggesting a hollow structure assembled *via* narcissistic face-to-face interactions, similar to the one observed for α-helices nanoparticles.^24^1Zn-Bz_EtOH_ also presented nanoparticles when analysed using AFM, however, the nanoparticles diameter range was considerably larger than observed for the methanol sample (Fig. 2d and SI13†). While the maximum height observed was still suggesting flattened particles, we found a correlation between diameter of the nanoparticle and its thickness (Fig. 2c). This was in contrast with 1Zn-Bz_MeOH_ for which the particles thickness was independent from their size. We believe that these differences are likely due to 1 presenting both polyproline I and II conformations in ethanol-triethyl amine as evidenced by the CD study (Fig. SI18†), likely impacting on the desired metal driven assembly. Finally, as for the other two samples, 1Zn-Bz_PrOH_ was prepared and analysed by DLS and AFM. We anticipated that 1 in the polyproline I form (*i.e.* in propanol) will still be able to assemble into metallo-peptide nanoparticles as this helical from, despite having a different chirality compared to the polyproline II, still presents a similar topology to an α-helix (Fig. 1a). DLS analysis for this sample showed the presence of nanoparticles of average size 205.3 nm and with an average PdI of 0.119 (Fig. SI6†). Analysis of this sample by AFM, presented nanoparticles with a diameter ranging from 140 nm to 190 nm (Fig. 2e and SI14†). The particles showed maximum heights between 2 nm and 4 nm, consistent with hollow, flattened nanoparticles. The difference in thickness between 1Zn-Bz_PrOH_ and 1Zn-Bz_MeOH_ could be attributed to the reduced length of 1 in the polyproline I form (*i.e.*1 would be 1.6 time shorter in the polyproline I form). Transmission electron microscopy (TEM) analysis of these samples was performed. As the samples contained zinc metal ions, we analysed them without any staining. All samples contained the expected morphologies with nanoparticles of similar sizes as observed *via* AFM (Fig. 2b, d and f). To the best of our knowledge this is the first example of a polyproline peptide divergent supramolecular assembly into nanoparticles. The polydispersity observed for some of these samples is probably due to the introduction of defects in the supramolecular network. In fact, while the design of 1 aims to promote a narcissistic assembly between different peptides (*i.e.* with the same faces), there are still two possible orientations the peptides could take while assembling; one in which the two polyprolines are parallel to each other and one in which they are antiparallel. We believe these two possible approaches will have an impact on the supramolecular assembly of 1 and its polydispersity. To test our hypothesis that narcissistic facial recognition and self-sorting was crucial to assembly of 1 into nanoparticles, peptide 2 was synthesised. Polyproline 2 similarly to 1 has two carboxylic acids on each face, however, in this peptide the two carboxylic acid groups are equidistant on all faces (Fig. 3).

We anticipated that the peptide 2 sequence will prevent the same degree of self-sorting during supramolecular assembly, drastically impacting the morphology obtained, hence supporting that the peptide 1 sequence is driving the controlled assembly into nanoparticles. Peptide 2 was dissolved in methanol and propanol, and incubated with and without triethylamine. CD analysis was performed and, surprisingly we found that 2 in propanol did not switch into polyproline I but retained a stable polyproline II conformation (Fig. SI19 and SI20†). This was the first indication of the effect of sequence editing 1. When AFM analysis was performed for 2Zn_MeOH_ and 2Zn_PrOH_ no nanoparticles could be identified for these samples. In the case of 2Zn_MeOH_, short worm-like metallo-peptide structures were found, while for 2Zn_PrOH_ a ridged continuous film was found (Fig. 3). AFM of peptide 1 and 2 in all solvents and triethylamine were recorded as a control experiment and no nanoparticles or other morphologies could be identified in these samples (See ESI†). These results support our hypothesis that the polyproline sequence play a key role in this controlled divergent supramolecular assembly process.

## Conclusions

In conclusion, we have demonstrated that the unique ability of polyproline peptides to interconvert into two stable and distinct secondary structures, can be used to access divergent supramolecular assemblies. We have shown that akin to other secondary structures used in supramolecular chemistry, the sequence of polyproline peptides can be rationally engineered without compromising their stability or periodicity, properties crucial in the rational design of novel supramolecular constructs. This work takes advantage of the unique properties associated with the polyproline secondary structures and uses them as powerful supramolecular building blocks. Our aim is to further expand the toolbox of structured peptides available to the community to assemble complex and responsive bioinspired supramolecular materials. Future work within our team, will focus on new polyproline systems capable of self-assembly in aqueous environments with potential applications in molecular transport and will be reported in due time.

## Data availability

The data supporting this article have been included as part of the ESI† file available online.

## Author contributions

AP, DFB, performed the synthesis purification and data analysis for all species synthesized herein. DFB and KS performed the AFM studies and analysis. JAW and MWF performed the TEM studies and data analysis for this work. The manuscript was written through contributions of all authors. All authors have given approval to the final version of the manuscript.

## Conflicts of interest

There are no conflicts to declare.

## Supplementary Material

NA-007-D4NA00762J-s001
