# Supplementary material for: Sequence-controlled divergent supramolecular assembly of polyproline helices into metallo-peptide nanoparticles
Source: Nanoscale Adv. 2024 Dec 5;7(1):94–8. doi: 10.1039/d4na00762j (PMC11626207; doi:10.1039/d4na00762j)
Supplement: NA-007-D4NA00762J-s001 [file NA-007-D4NA00762J-s001.pdf]

Supplementary Information (SI) for Nanoscale Advances.
This journal is © The Royal Society of Chemistry 2024

Supporting Information

Sequence-Controlled Divergent Supramolecular Assembly of Polyproline Helices into Metallo-Peptide Nanoparticles

Dominic F. Brightwell,[b] Kushal Samanta,[c] Julie A. Watts,[d,e] Michael W. Fay,[d,f] and Aniello Palma*[a]

[a] *School of Chemistry, University College Dublin, Belfield, Dublin 4, Ireland.*

[b] *School of Chemistry and Forensic Science, Supramolecular and Interfacial Chemistry, Ingram building, The University of Kent, Canterbury CT2 7NZ, Kent, United Kingdom*

[c] *School of Chemistry, University of Birmingham, Edgbaston, Birmingham, B15 2TT, UK.*

[d] *Nanoscale and Microscale Research Centre, University of Nottingham, Nottingham, NG7 2RD, UK.*

[e] *School of Pharmacy, University of Nottingham, Nottingham, NG7 2RD, UK.*

[f] *Faculty of Engineering, University of Nottingham, Nottingham, NG7 2RD, UK.*

Corresponding author: aniello.palma@ucd.ie

Abstract: The field of peptide based supramolecular biomaterials is fast evolving. These types of constructs have been shown to find applications in the fields of bioimaging, drug delivery and scaffolds for chemical reactions. However, the community typically focuses on the use of two specific class of structured peptides: α-helices and β-sheets, clearly neglecting a unique peptide secondary structure: the polyproline helix. Herein, we report the first design, synthesis and characterization of polyproline based metallo-peptide nanoparticles. We demonstrate that rationally engineered polyproline helices can assemble in a divergent manner, into two types of nanoparticles. We also demonstrate that the primary sequence of the functionalised polyproline peptide, is crucial to ensure a controlled assembly. This work clearly demonstrates that polyproline helices can be a powerful tool to achieve supramolecular assemblies of complex and responsive bioinspired nanomaterials.

Table of Contents

Instruments and Methods..3

Monomer Synthesis 3

Synthesis of tosylate benzyl-(2S,4R)-4-hydroxypyrrolidine-2-carboxylate - **S1**: 4

Synthesis of benzyl-1-(2S,4R)-4-hydroxypyrrolidine-1-carboxylate-2-carboxylic acid - **S2**: 4

Synthesis of dibenzyl (2S,4R)-4-hydroxypyrrolidine-1,2-dicarboxylate – **S3**: 4

Synthesis of dibenzyl (2S,4R)-4-(2-(tert-butoxy)-2-oxoethoxy)pyrrolidine-1,2-dicarboxylate – **S4**:...5

Synthesis of (2S,4R)-4-(2-(tert-butoxy)-2-oxoethoxy)pyrrolidine-2-carboxylic acid - **S5**:6

Synthesis of (2S,4R)-1-(((9H-fluoren-9-yl)methoxy)carbonyl)-4-(2-(tert-butoxy)-2-oxoethoxy)pyrrolidine-2-carboxylic acid - **S6**: 6

Peptides Synthesis...12

Synthesis of Ac-$(P)_2XPXPX(P)_2(X)_3P$-NH_2- **1**: 13

Complexation reactions and nanoparticles characterisation:.....................................16

Peptide **1** and **2** metal complexation reaction: 23

CD Spectroscopy: 24

Instruments and Methods

HPLC: Semi Preparative HPLC was performed on a 1260 Infinity II (Agilent) HPLC, equipped with a C18 (Kormasil 100-5-C18, 10 x 250 mm) column at a 4.73 mL/min flow rate monitored at 205/225/254 nm wavelengths.

LC-MS: Compounds were separated via RP-HPLC using a HiChrom KR100 5C18 5263 column at 40 °C on a Dionex UltiMate 3000. Gradient: 5% B for 5 minutes then from 5% B to 100% B over 20 minutes, and held at 100% B for 5 minutes. Where A is Water (0.1 % formic acid) and B is methanol (0.1 % formic acid). Flow rate is 1.0 ml/min. Wavelength: 225 nm. The flow was directed into directed into the electrospray source of a Thermo Scientific MSQ Plus Mass Detector, operating in positive ion mode, at 75 kV and mass spectra recorded from 100-2000 m/z.

LC-HRMS: Spectra were recorded on an Agilent 6546 Q-TOF system equipped with an Agilent 1260 Infinity prime II LC inlet in positive electrospray mode *via* an Agilent Jetstream ESI source. MS data was processed using MassHunter software. Chromatography was effected *via* a 50mm Agilent Poroshell 2.7um C18 LC column, and mobile phases of 0.1% Formic Acid vs ACN on a gradient from 90% aq to 90% ACN over 5min with an isocratic hold of 5min at 90% ACN. ESI Source conditions were as follows: fragmentor voltage set to 175V, Drying Gas temperature of 325C and Sheath Gas temperature of 350C. Capillary voltage was 4000V and nozzle voltage 2000V.

NMR spectroscopy. 1D and 2D NMR spectra were collected either on a Bruker Avance II 400 MHz spectrometer, a Bruker Avance III 400 MHz at 298 K. Chemical shifts are with reference to the residual solvent peak, with J values in Hz. For multiplicity of the peaks, the abbreviations used are (s) singlet, (d) doublet, (t) triplet, (q) quartet and (m) multiplet.

Monomer Synthesis

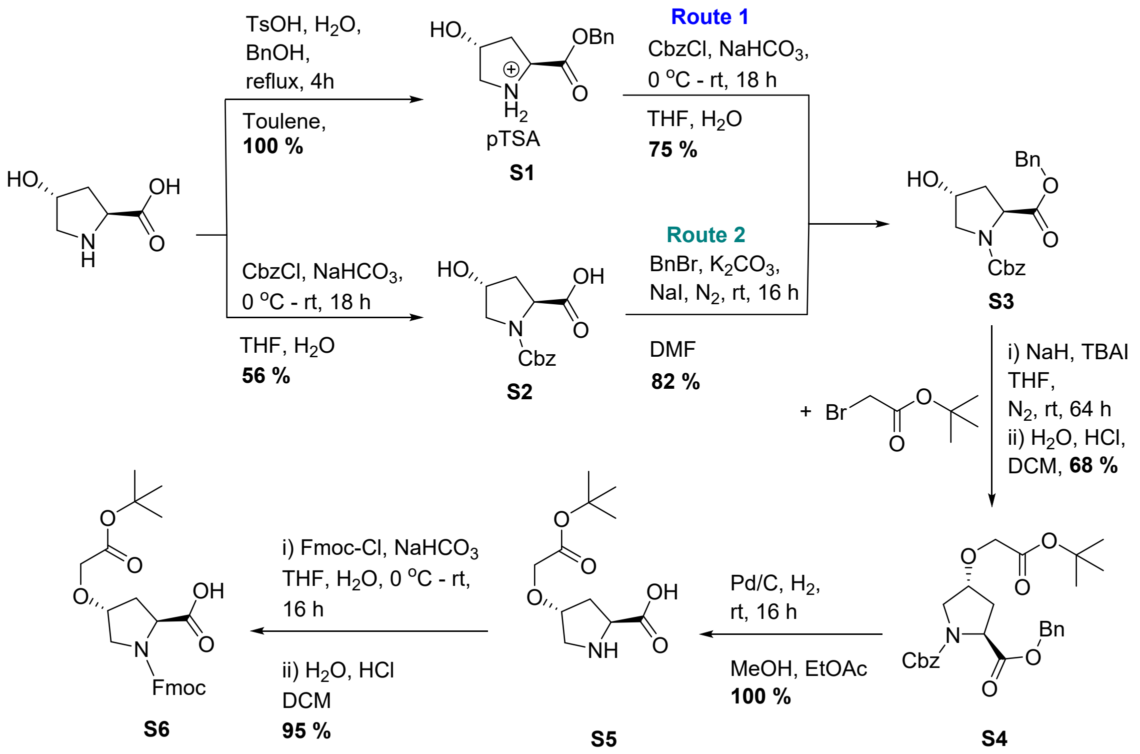

Scheme SI1. Synthesis of molecule **S6**. Compound **S3** was obtained using two independent synthetic strategies as reported in this scheme.

Synthesis of tosylate benzyl-(2S,4R)-4-hydroxypyrrolidine-2-carboxylate - **S1**:

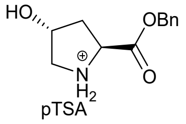

S1

Following the method reported in *Journal of Fluorine Chemistry 129 (2008) 286–293;* Trans-L-Hyp-OH, (1.00 g, 7.63 mmol, 1 eq) was dissolved in toluene (10 ml). Benzyl alcohol (3.2 ml, 30.5 mmol, 4 eq) was then added to this solution with p-TsOH.H_2O (1.17 g, 9.156 mmol, 1.2 eq), the reaction was then heated at reflux for 4 h. The solution was then diluted with Et_2O (20 ml) and the resulting precipitate was isolated via filtration and washed with Et_2O to yield the product, **S1**, as a white foam in a quantitative yield, this was used as is for the next step.

Synthesis of benzyl-1-(2S,4R)-4-hydroxypyrrolidine-1-carboxylate-2-carboxylic acid - **S2**:

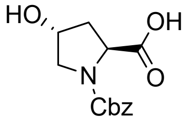

S2

Trans-L-Hyp-OH, (7.0125 g, 53.48 mmol, 1 eq) was dissolved aq. NaHCO3 (30 ml). The resulting solution was stirred at 0 °C for 30 minutes before adding Cbz-Cl (9.2 ml, mmol, 1.2 eq). The solution was stirred at 0 °C for 1h and then stirred for 18 h at rt. The reaction was monitored via TLC (6:4, Hex/EtOAc). Upon completion the reaction was diluted with deionised water (100 ml) and washed with cold Et_2O (x 3) while at pH 8. The solution was then acidified to pH 2 with 3 M HCl and extracted with EtOAc (x 3). The combined EtOAc extracts were then washed with brine (x 2), dried over anhydrous $MgSO_4$, filtered and the solvent was removed under vacuo to yield **S2** as an oil (7.923 g, mmol, 56 %). ^{1}H NMR (400 MHz, MeOD) δ 7.39 – 7.25 (m, 5H, Cbz), 5.16 – 5.03 (m, 2H, Cbz), 4.47 – 4.36 (m, J = 16.0, 8.0 Hz, 2H), 3.65 – 3.49 (m, 2H), 2.38 – 2.23 (m, J = 12.9, 9.7, 8.1, 2.9, 1.5 Hz, 1H), 2.16 – 2.01 (m, 1H). ^{13}C NMR (101 MHz, MeOD) δ 176.26, 175.97, 156.84, 156.52, 137.87, 137.73, 129.44, 129.07, 128.87, 128.55, 70.65, 69.96, 68.28, 59.29, 59.02, 56.02, 55.65, 40.16, 39.31. LCMS (ESI-MS) m/z: $[M+Na]^+$ calcd for $C_{13}H_{15}NO_5$ = 288.08, found = 288.00.

*Synthesis of dibenzyl (2S,4R)-4-hydroxypyrrolidine-1,2-dicarboxylate – **S3**:*

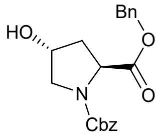

S3

Route 1: 4-*trans*-L-Hydroxyproline benzyl ester tosylate salt, **S1**, (8.44g, 21.5 mmol, 1 eq) and $NaHCO_3$ (4.52 g, 53.8 mmol, 2 eq) were dissolved in water (32 mL) and after 5 min acetone (32 mL) was added followed by Cbz-Cl (3.2 ml, 22.5 mmol, 1.1 eq). The resulting slurry was stirred overnight at room temperature. The reaction mixture was quenched adjusting to pH 1-2 with 3 M HCl and diluted with H_2O (300 ml), the solution was then extracted with Et_2O (3 x 350 mL). The combined organic extracts were washed with saturated aqueous $NaHCO_3$ (250 mL) and 5 % aqueous NaCl (150 mL), dried over $MgSO_4$, filtered, and concentrated on a rotary evaporator. The residue was purified by flash chromatography (FCC) (3:2 Hex/EtOAc) to give the title compound, **S3** (5.69 g, 16.13 mmol, 75 %), as a clear and colourless oil.

Route 2: Compound **S2** (7.92 g, 29.87 mmol, 1 eq), K_2CO_3 (9.10 g, 65.71 mmol, 2.2 eq) and NaI (0.45 g, 2.99 mmol, 0.1 eq) were dissolved in DMF (22 ml) under a N_2 atmosphere. BnBr (8.82 ml, 89.6 mmol, 3 eq) was added dropwise over 5 mins to the stirring solution. The solution was stirred overnight at rt forming a yellow solution with a white precipitate. The reaction was monitored via TLC (3:2, Hex/EtOAc). The reaction was diluted with EtOAc and washed with deionised water (x 4) and brine (x 4), before drying over anhydrous MgSO4 and filtering. The solvent was removed under vacuo to yield an orange oil, this was then washed with hexane (x 5) forming a pink extract. The washed oil was then purified via FCC (3:2, Hex/EtOAc) and concentrated to yield a yellowish oil, **S3** (8.68 g, 24.4 mmol, 82 %).^{1}H NMR (400 MHz, $CDCl_3$) δ 7.39 – 7.16 (m, 10H), 5.25 – 4.94 (m, 4H), 4.55 (dt, *J* = 15.6, 7.9 Hz, 1H), 4.45 – 4.34 (m, 1H), 3.68 – 3.49 (m, 2H), 3.08 (s, 1H), 2.35 – 2.21 (m, 1H), 2.08 – 1.97 (m, *J* = 8.1, 7.2, 4.7 Hz, 1H). ^{13}C NMR (101 MHz, $CDCl_3$) δ 172.68, 172.48, 155.18, 154.71, 136.40, 136.19, 135.57, 135.36, 128.57, 128.49, 128.44, 128.38, 128.29, 128.15, 128.09, 128.05, 128.01, 127.85, 127.78, 69.88, 69.15, 67.30, 67.27, 66.98, 66.85, 60.52, 58.14, 57.90, 55.23, 54.63, 39.11, 38.31, 21.06, 14.19.

*Synthesis of dibenzyl (2S,4R)-4-(2-(tert-butoxy)-2-oxoethoxy)pyrrolidine-1,2-dicarboxylate – **S4**:*

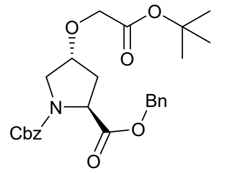

S4

NaH, 60 % in mineral oil, (1.26 g, 31.62 mmol, 2.5 eq) was placed in an oven dried flask under a N_2 atmosphere. Dry THF (30 ml) was then added slowly. Tert-butyl bromoacetate (7.46 ml, 50.50 mmol, 4 eq) and TBAI (3.75 g, 10.2 mmol, 0.8 eq) in dry THF (5 ml) was then added in one portion. The solution was stirred at rt for 20 mins. Compound **S3** (4.49 g, 12.6 mmol, 1 eq) in dry THF (18 ml) was added dropwise over 2 h. This formed an off-white cloudy solution and was left stirring at rt for 64 h hours. TLC analysis (6:4, Hex/EtOAc) was carried out to confirm reaction completion. The reaction was then diluted with deionised water (200 ml), acidified to pH 6-7 with 3 M HCl and quickly extracted with DCM (150 ml x 3). The combined organic layers were then washed with brine and dried over anhydrous $MgSO_4$. The remaining solvent was then removed under vacuo. The crude yellow oily product (16.57 g) was then placed under high vac at 65 °C with a nitrogen trap, to remove residual tert-butyl bromoacetate yielding an oily partially yellow crystalline crude product (10. 9579 g). This was then purified by FCC (3:1, Hex/EtOAc), yielding compound **S4** as an oil (4.0g, 8.6 mmol, 68 %).Spectral Data ^{1}H NMR (400 MHz, $CDCl_3$) δ 7.53 – 7.37 (m, 9H), 7.37 – 7.31 (m, 1H), 5.38 - 5.28 (m, 1H), 5.27 (s, 1H), 5.18 (s, 1H), 5.13 (s, 1H), 4.67 (dt, 1H), 4.37 – 4.31 (m, 1H), 4.08 (dt, 2H), 3.95 – 3.72 (m, 2H), 2.66 – 2.49 (m, 1H), 2.29 – 2.18 (m, 1H), 1.60 (d, *J* = 3.4 Hz, 9H, *t*Bu). ^{13}C NMR (101 MHz, $CDCl_3$) δ 172.49, 172.26, 169.18, 154.86, 154.34, 136.49, 136.37, 135.64, 135.42, 128.59, 128.50, 128.44, 128.37, 128.30, 128.14, 128.09, 128.05, 127.98, 127.93, 127.85, 82.00, 78.00, 77.26, 67.26, 67.20, 67.01, 66.96, 66.92, 66.82, 58.10, 57.86, 51.90, 51.79, 36.77, 35.48, 28.11.

*Synthesis of (2S,4R)-4-(2-(tert-butoxy)-2-oxoethoxy)pyrrolidine-2-carboxylic acid - **S5**:*

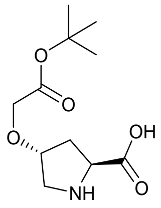

S5

Pd/C 5 % (0.20 g, 20 % w/w) was placed in a three-neck flask with stirrer bar under a N_2 atmosphere. The catalyst was then covered with ethyl acetate (10 ml) and methanol (10 ml) was then added in a stream down the side of the flask. Compound **S4** (0.80 g, 1.7 mmol, 1 eq) in methanol (10 ml) was then added. Stirring was then started and the flask was evacuated till the solvent was bubbling and refilled with nitrogen three times. The vessel evacuated and exposed to a H_2 atmosphere with a H_2 balloon. The balloon was then left open to the flask with the needle underneath the solvent line and the reaction stirred overnight. The hydrogen balloon was refilled as needed. The reaction was monitored via TLC (8:2, Hex/EtOAc). After completion of the reaction the solution was filtered through a celite filter and the solvent removed under vacuo to yield compound **S5** as a white crystalline solid (0.42 g, 1.7 mmol, 100%). Spectral Data: ^{1}H NMR (400 MHz, MeOD) δ 4.39 – 4.29 (m, 1H, α H), 4.20 – 4.13 (m, 1H), 4.13 – 3.97 (m, 2H), 3.50 – 3.35 (m, 2H), 2.64 – 2.51 (m, 1H), 2.11 – 2.00 (m, 1H), 1.48 (d, *J* = 4.3 Hz, 9H, *t*Bu). ^{13}C NMR (101 MHz, MeOD) δ 173.66, 171.49, 83.19, 80.33, 67.60, 61.36, 52.10, 36.34, 28.30.

*Synthesis of (2S,4R)-1-(((9H-fluoren-9-yl)methoxy)carbonyl)-4-(2-(tert-butoxy)-2-oxoethoxy)pyrrolidine-2-carboxylic acid - **S6**:*

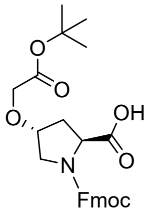

S6

Compound **S5** (1.43 g, 5.83 mmol, 1 eq) was dissolved in THF (40 ml) and saturated aq. $NaHCO_3$ (40 ml) in an ice bath. Fmoc-Cl (1.81 g, 7.00 mmol, 1.2 eq) was dissolved in THF (7 ml) and added to the solution at 0 °C. The pH was checked after 1h and adjusted with excess $NaHCO_3$ to pH 8. The solution was then left stirring overnight at rt. The reaction was monitored via TLC (6:4, Hex/EtOAc). The reaction was quenched with ice cold water (250 ml) and washed with cold Et_2O (150 ml x 3). The aqueous solution was then acidified to pH 2 with 3 M HCl and quickly extracted with DCM (500 ml x 3). The combined DCM layers were then washed with brine (x 2), dried over anhydrous $MgSO_4$, filtered, and the solvent removed under vacuo to yield a white crystalline solid, **S6** (2.59 g, 5.54 mmol, 95 %). Spectral Data: ^{1}H NMR (400 MHz, $CDCl_3$) δ 7.76 (d, *J* = 7.5 Hz, 1.25H), 7.69 (d, *J* = 7.5 Hz, 0.75H), 7.60 – 7.50 (m, 2H), 7.43 – 7.23 (m, 4H), 6.57 (s, 1H), 4.56 – 4.29 (m, 3H), 4.28 – 4.09 (m, 2H), 4.02 – 3.86 (m, 2H), 3.81 – 3.57 (m, 2H), 2.57 –2.37 (m, 1H, two rotamers), 2.30 – 2.11 (m, 1H, two rotamers), 1.48 (s, 9H, two rotamers). ^{13}C NMR (101 MHz, $CDCl_3$) δ 177.21, 175.54, 169.34, 155.95, 154.69, 144.07, 143.82, 143.78, 141.40, 141.38, 141.32, 127.87, 127.74, 127.23, 127.18, 125.19, 125.05, 120.11, 120.00, 82.30, 77.85, 68.13, 68.04, 67.90, 67.05, 66.99, 58.14, 57.48, 51.92, 51.83, 47.20, 47.15, 36.99, 34.98, 28.21, 28.18, 25.67. Mass spectrometry: ESI-MS(m/z):[M-Na]+ 490.10.

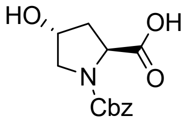

S2

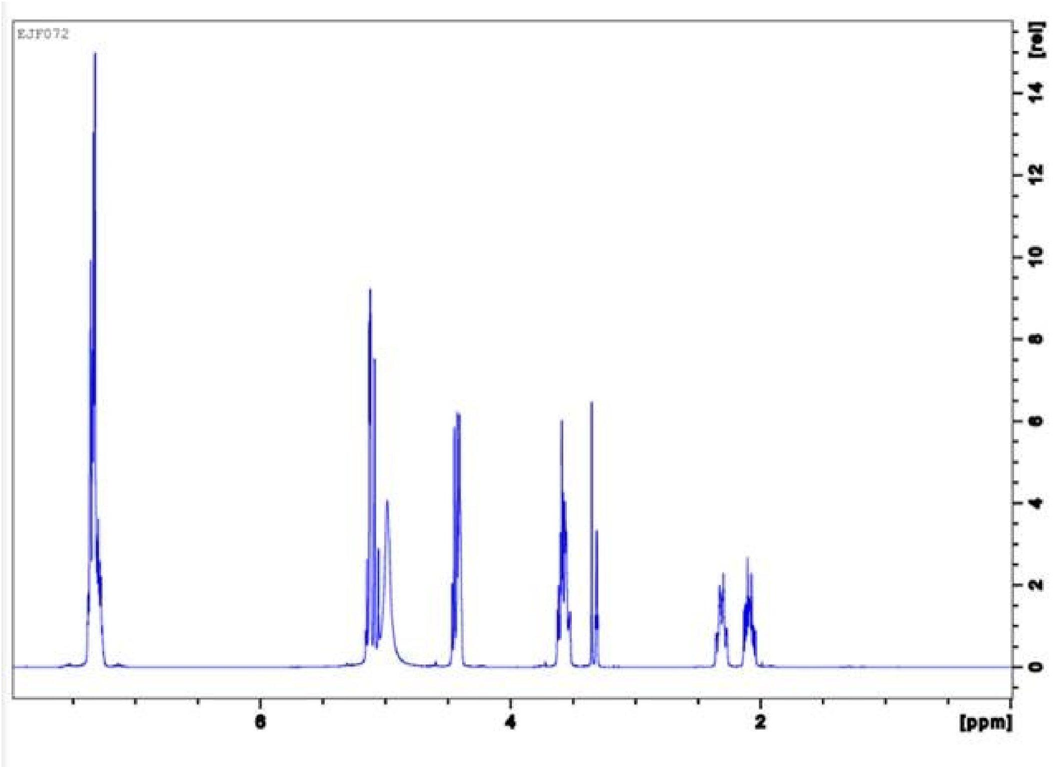

^{1}H NMR of **S2 (400 MHz, MeOD)**

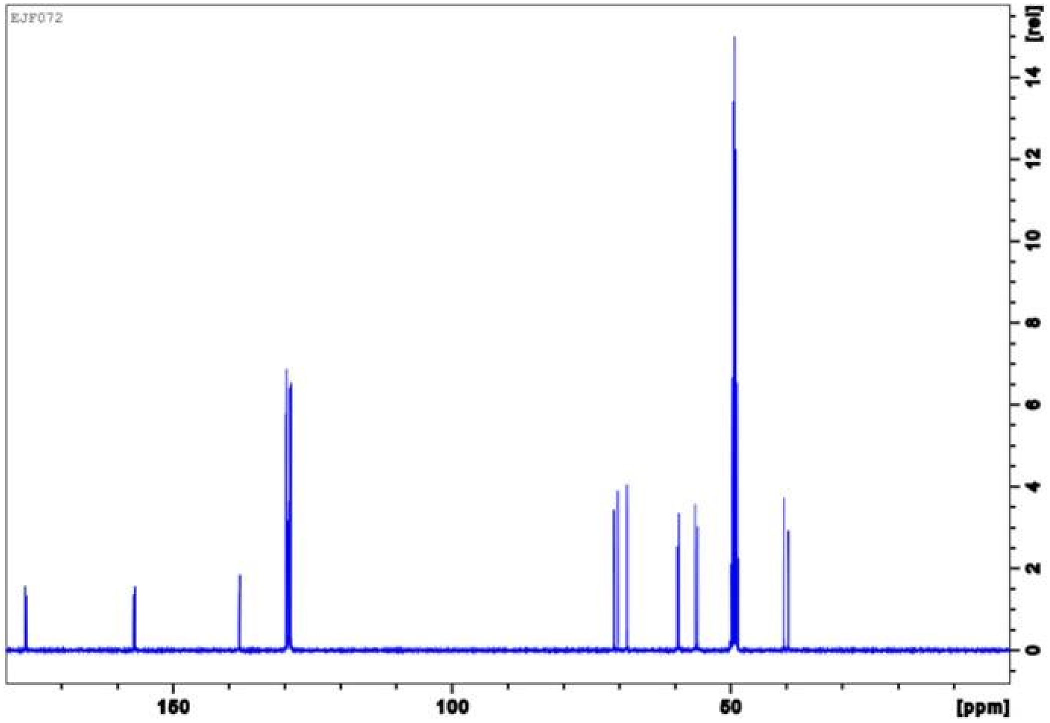

^{13}C NMR of **S2 (101 MHz, MeOD)**

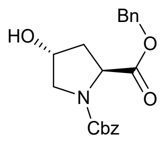

S3

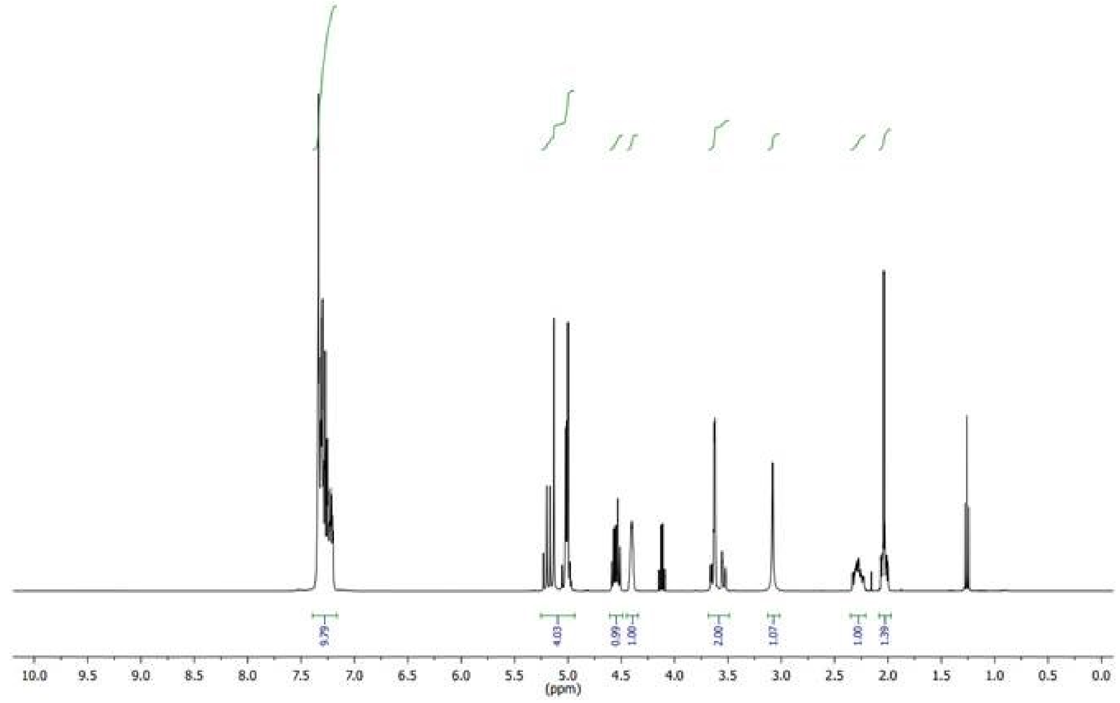

^{1}H NMR of **S3 (400 MHz, $CDCl_3$)**

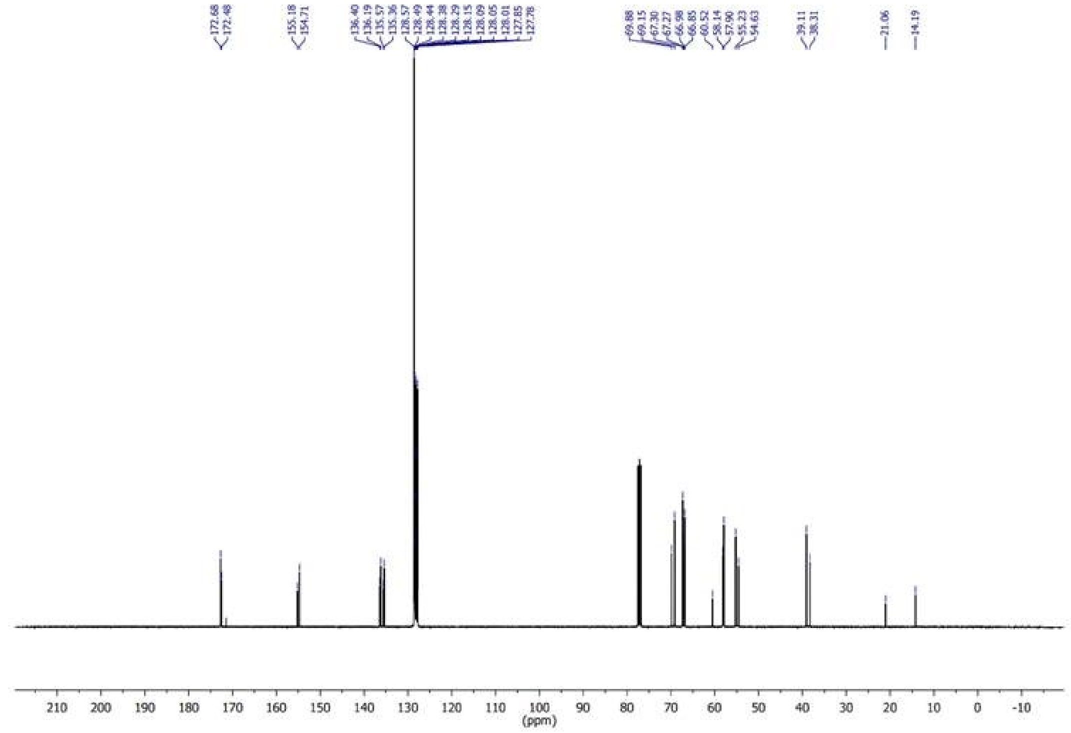

^{13}C NMR of **S3 (101 MHz, $CDCl_3$)**

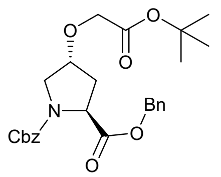

S4

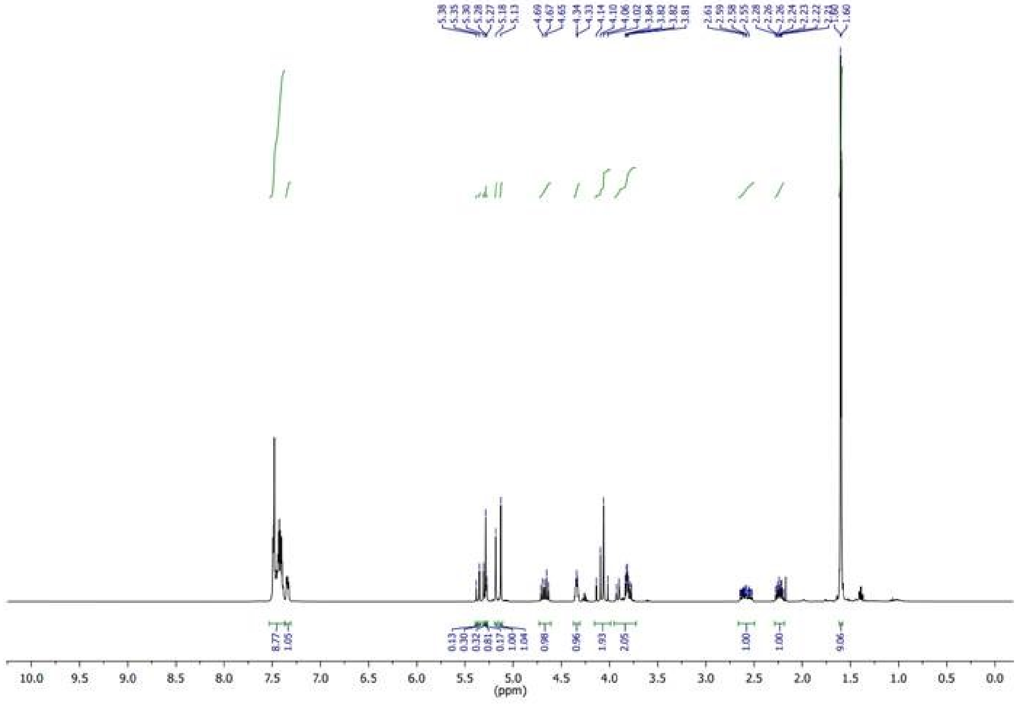

^{1}H NMR of **S4 (400 MHz, $CDCl_3$)**

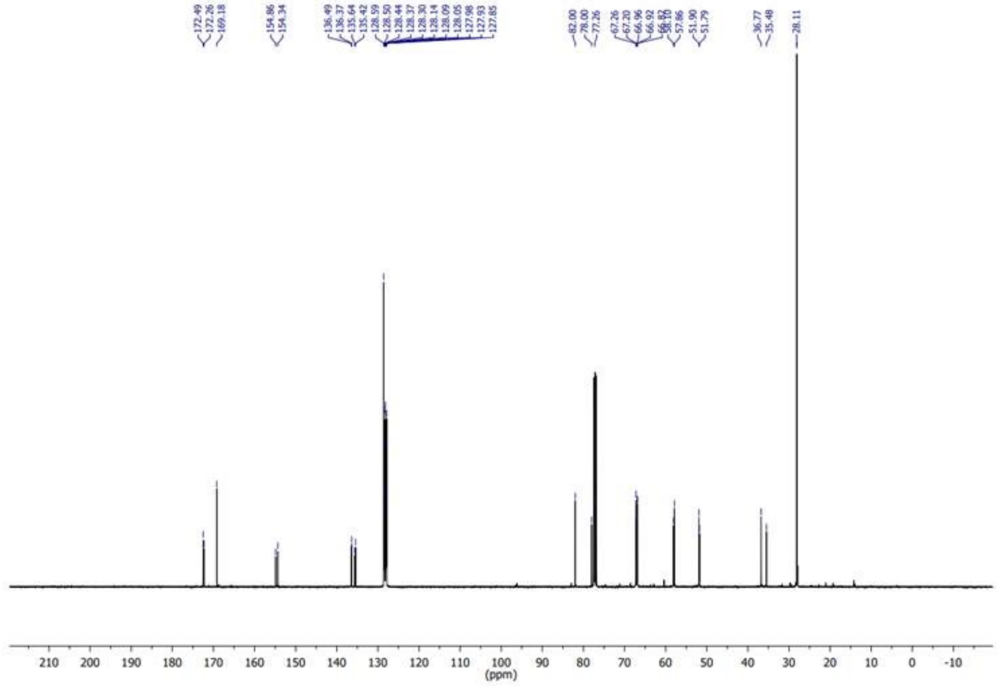

^{13}C NMR of **S4 (101 MHz, $CDCl_3$)**

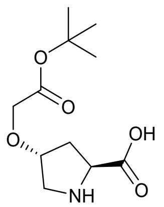

S5

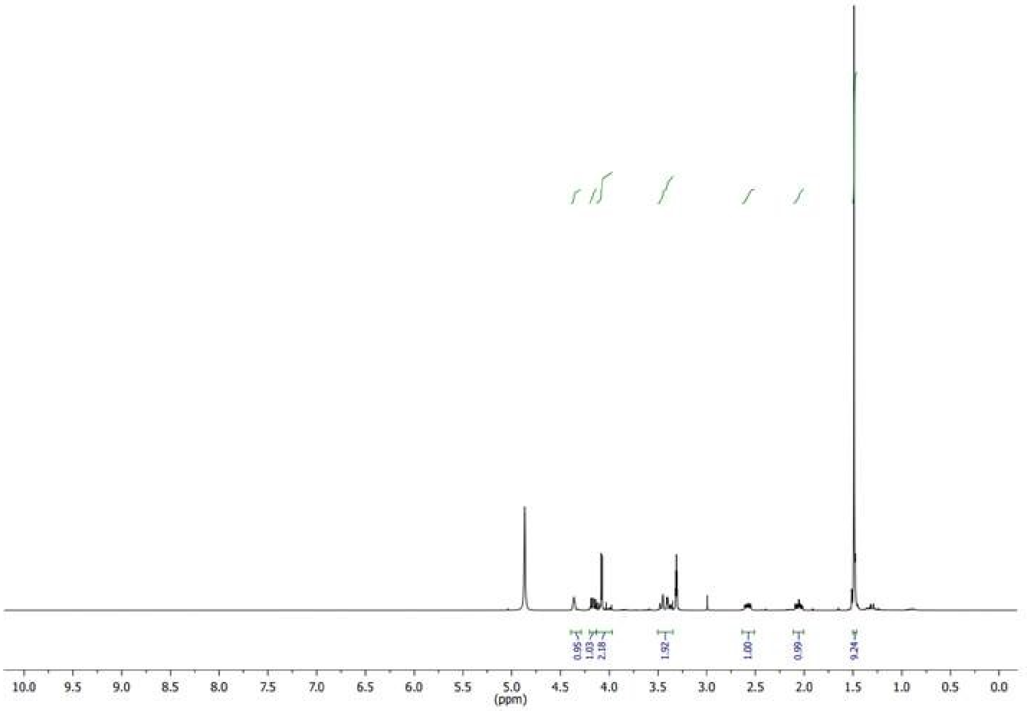

^{1}H NMR of **S5 (400 MHz, MeOD)**

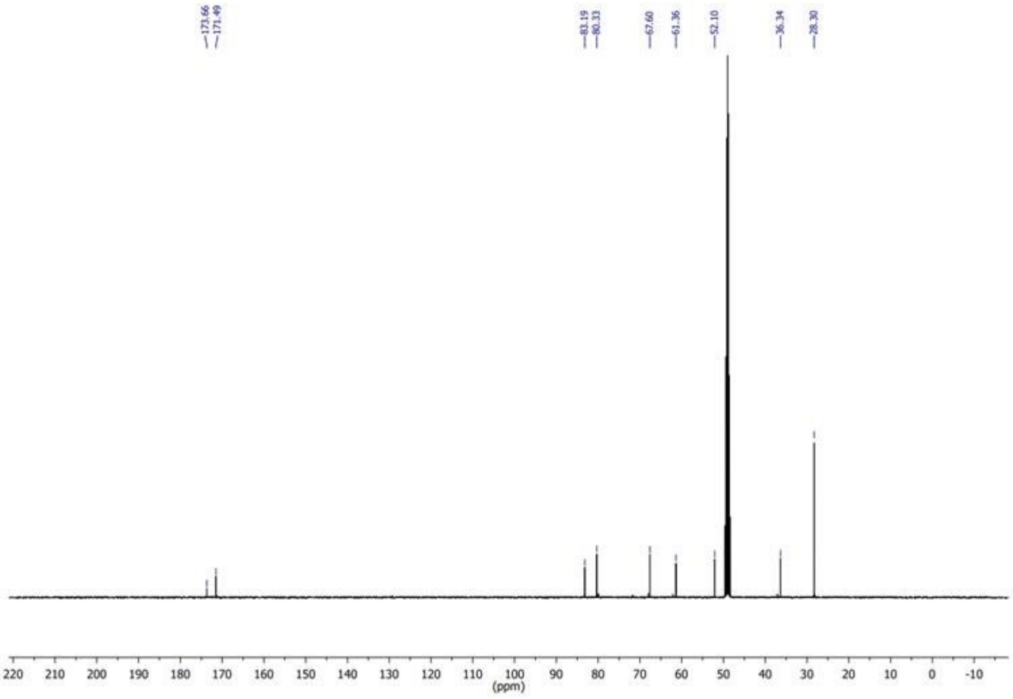

^{13}C NMR of **S5 (101 MHz, MeOD)**

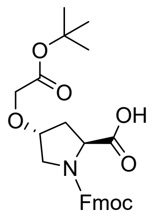

S6

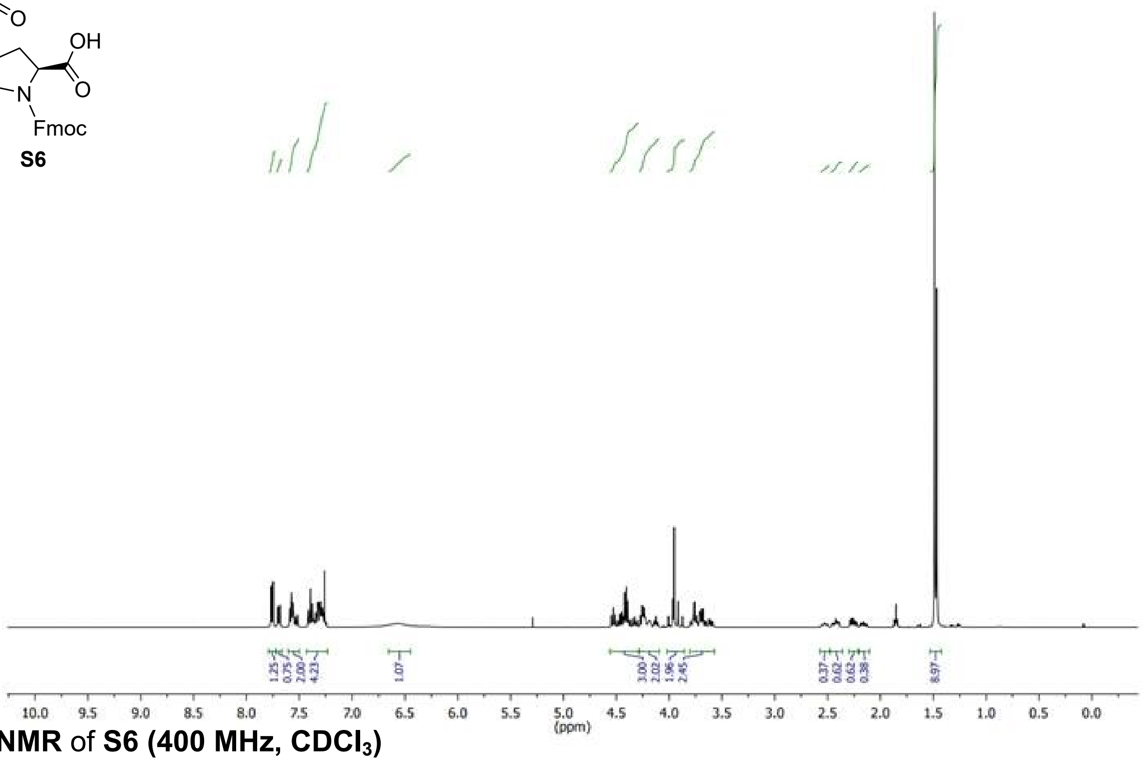

^{1}H NMR of **S6 (400 MHz, CDCl$_3$)**

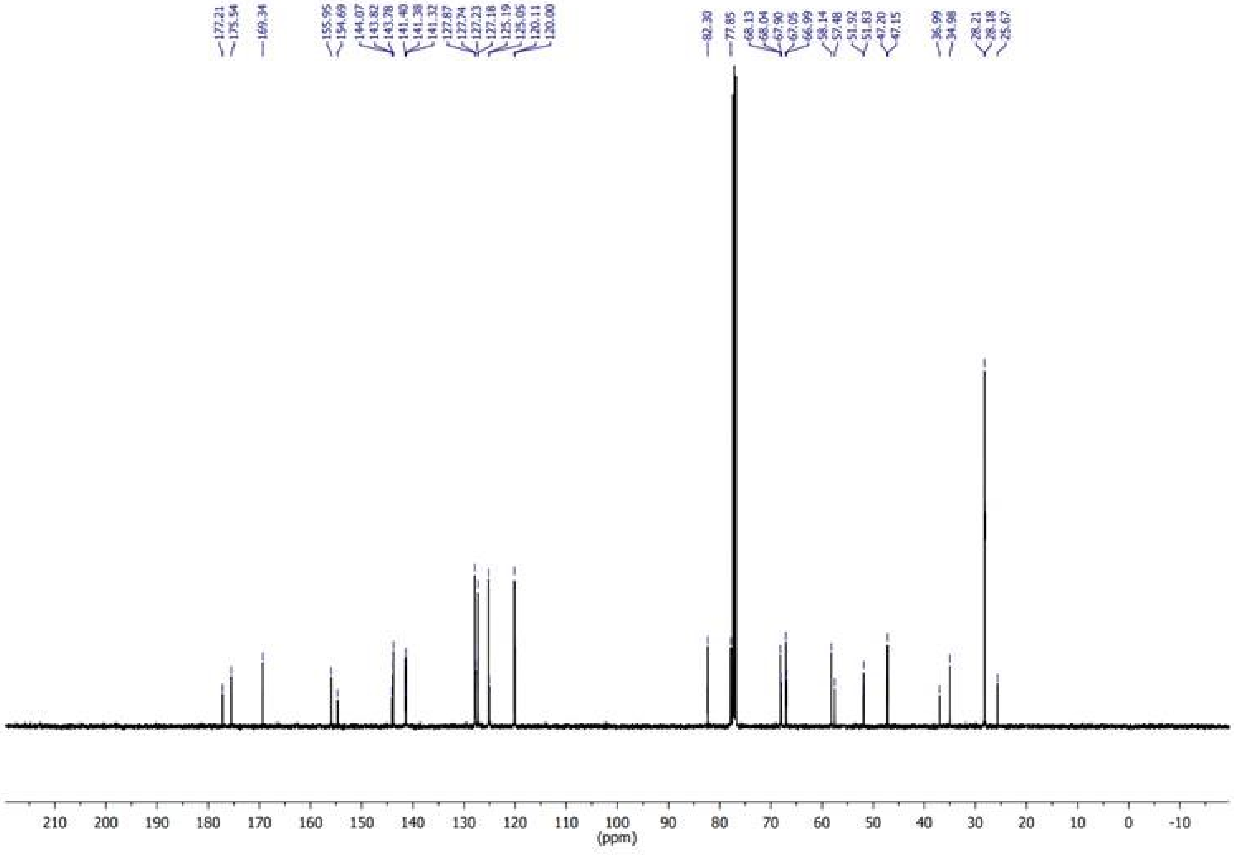

^{13}C NMR of **S6 (101 MHz, CDCl$_3$)**

Peptides Synthesis

Materials – Rink Amide MBHA resin (100-200 mesh, 0.3 mmol/g) 1% DVB, Fmoc-amino acids, N,N-Diisopropylethylamine (DIPEA), acetic anhydride (Acac), pivalic anhydride, Trifluoroacetic acid (TFA), Diisopropylcarbodiimide (DIC), Ethyl cyano(hydroxyimino)acetate (Oxyma Pure), and benzotriazol-1-yl-oxytripyrrolidinophosphonium hexafluorophosphate (PyBOP) were obtained from Fluorochem Ltd (Derbyshire, UK).

Peptide Synthesiser Method – All peptides were synthesised on a 0.1 mmol scale on Rink amide MBHA resin using a Liberty^{TM} microwave peptide synthesiser (CEM) utilising Fmoc solid-phase peptide synthesis techniques and repeated steps of single deprotections, and couplings interspaced with washings (4 x 4 ml DMF). The synthesis was paused after the final coupling step and the resin removed from the reaction vessel before stopping the synthesis (to prevent gradual loss of the Fmoc group). Deprotection: 20 % piperidine in DMF (4.5 ml) for 5 min with 30 W microwave irradiation at 90 ^oC. Coupling: Fmoc-amino acid (1.5 ml, 0.2 M, 3 eq.), DIC (1.2 ml, 0.5 M, 6 eq.), Oxyma Pure (0.6 ml, 0.5 M, 3 eq.) in DMF, and DMF (3 ml) for 5 min at 90 ^oC with 30 W microwave irradiation. **Capping procedure:** Resin washed with DMF (x 5) and suspended in DMF with Acac (50 eq) and DIPEA (50 eq). Resin agitated for 30 mins before filtering and washing the resin multiple times with DMF.

Peptide Cleavage - The resin was then washed with DCM (x 5), before the Fmoc-protected peptide was cleaved from the resin with TFA (95 % in DCM) for 1.5 h. The resin was then washed with the cleavage cocktail (x 2) and the filtrate was concentrated by evaporation before precipitation in cold Et_2O and centrifugation. The solution was then decanted and the solid repeatedly washed with cold Et_2O to isolate the peptide, as a white solid in a quantitative yield after drying under vacuum. Peptides were then purified via RP-HPLC, > 99 % purity by analytical reverse-phase HPLC.

HPLC: Semi Preparative HPLC was performed on a 1260 Infinity II (Agilent) HPLC, equipped with a C18 (Kormasil 100-5-C18, 10 x 250 mm) column at a 4.73 mL/min flow rate monitored at 205/225/254 nm wavelengths.

Synthesis of Ac-$(P)_2XPXPX(P)_2(X)_3P$-NH_2- **1**:

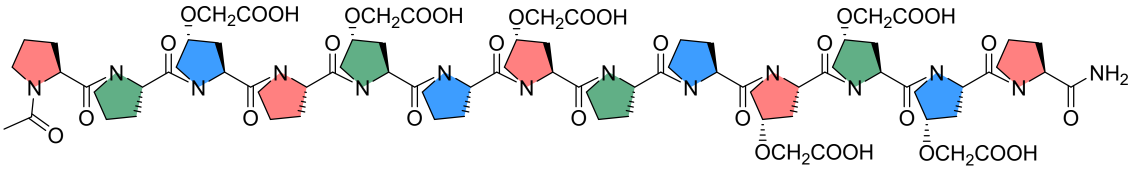

1

Peptide **1** was synthesised using standard SPPS techniques on 0.1 mmol scale using the standard Oxyma-DIC coupling method, quantitative yield. **1**, ^1H NMR (400 MHz, MeOD) δ 4.83 – 4.60 (m, 7H), 4.45 – 4.32 (m, 4H), 4.29 – 3.94 (m, 12H), 3.88 – 3.55 (m, 14H), 2.68 – 1.86 (m, 27H). HRMS: m/z calcd for $[M+2H]^{2+}$: $C_{79}H_{108}N_{14}O_{32}^+$, 883.3700; found; $[M+2H]^{2+}$ 883.3700

Synthesis of Ac-$X(P)_2(X)_2(P)_2(X)_2(P)_2XP$-$NH_2$ - **2**:

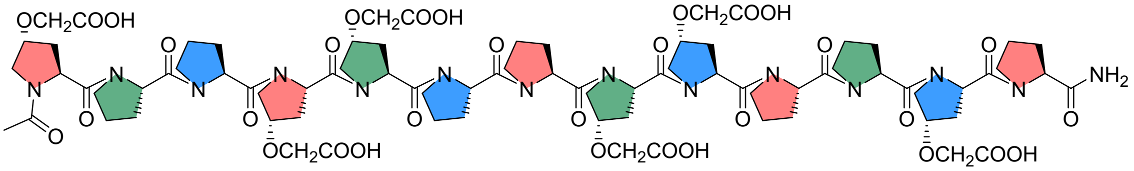

2

Peptide **2** was synthesised using standard SPPS techniques on 0.1 mmol scale using the standard Oxyma-DIC coupling method, quantitative yield. ^1H NMR (400 MHz, MeOD) δ 4.73 – 4.62 (m, 3H), 4.45 – 4.28 (m, 4H), 4.28 – 3.93 (m, 10H), 3.92 – 3.46 (m, 12H), 2.57 – 2.33 (m, 4H), 2.32 – 2.15 (m, 4H), 2.14 – 1.86 (m, 16H). HRMS: m/z calcd for $[M+2H]^{2+}$: $C_{79}H_{108}N_{14}O_{32}^+$, 883.3700; found; $[M+2H]^{2+}$ 883.3704

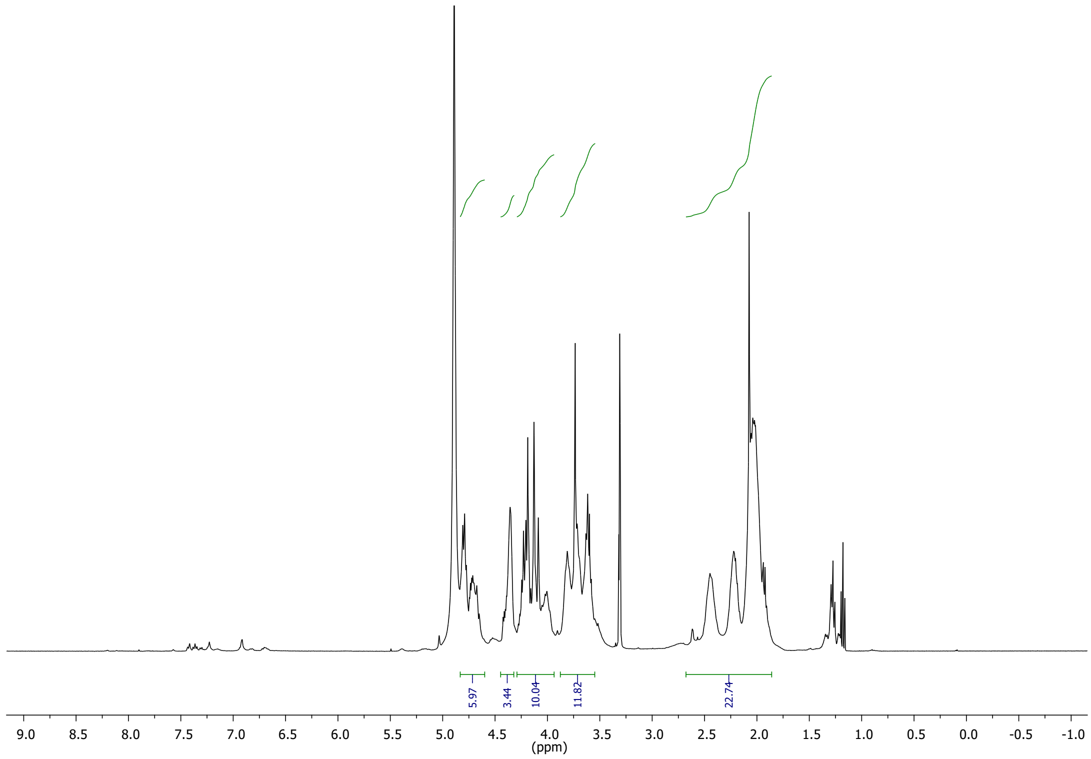

^1H NMR of **1** (400 MHz, MeOD).

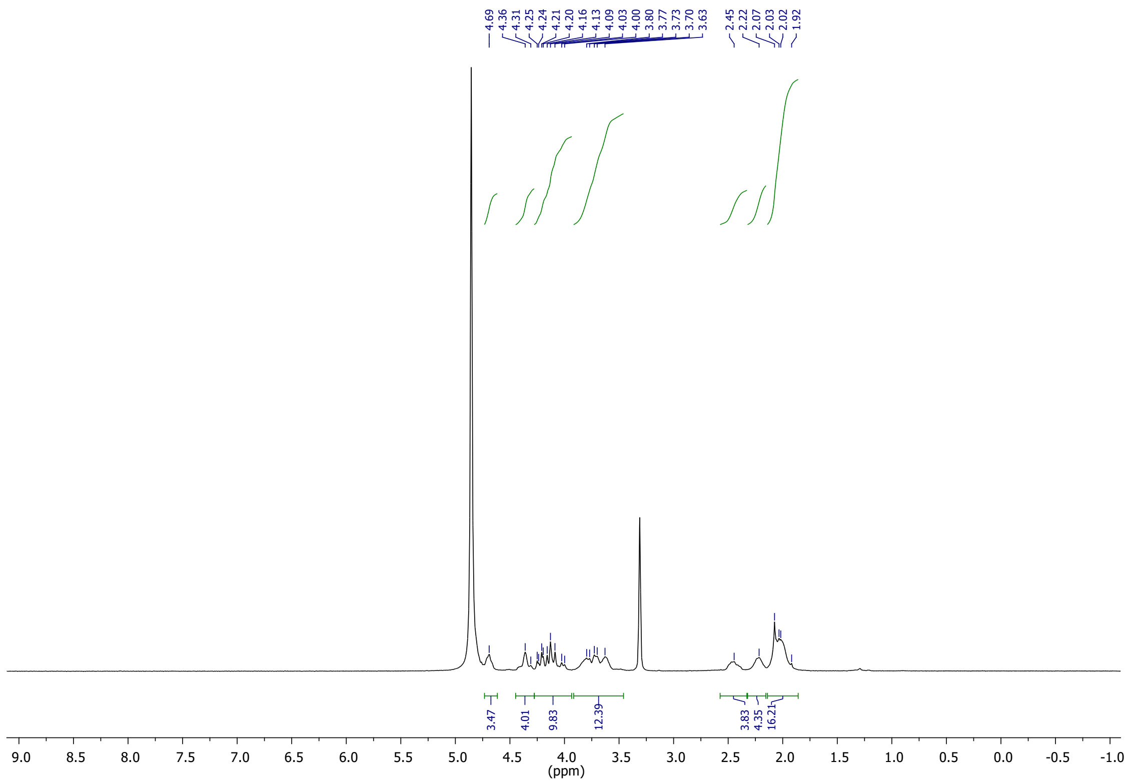

¹H NMR of peptide **2** (400 MHz, MeOD).

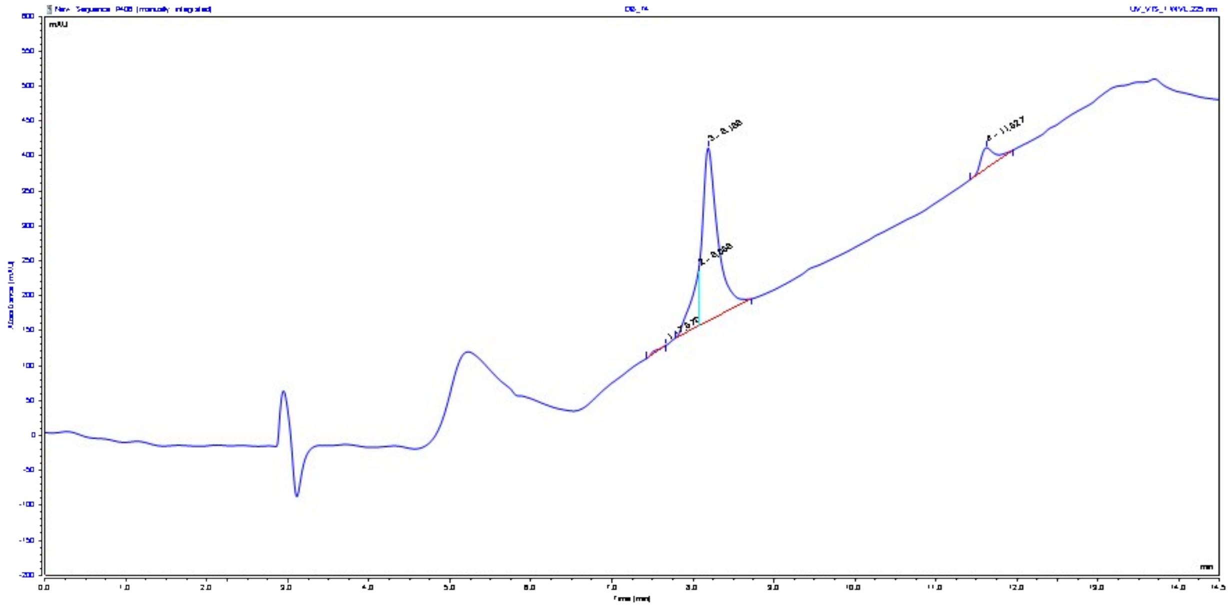

HPLC UV-Vis Spectrum of peptide **1**, rt = 8.188 min, 225 nm.

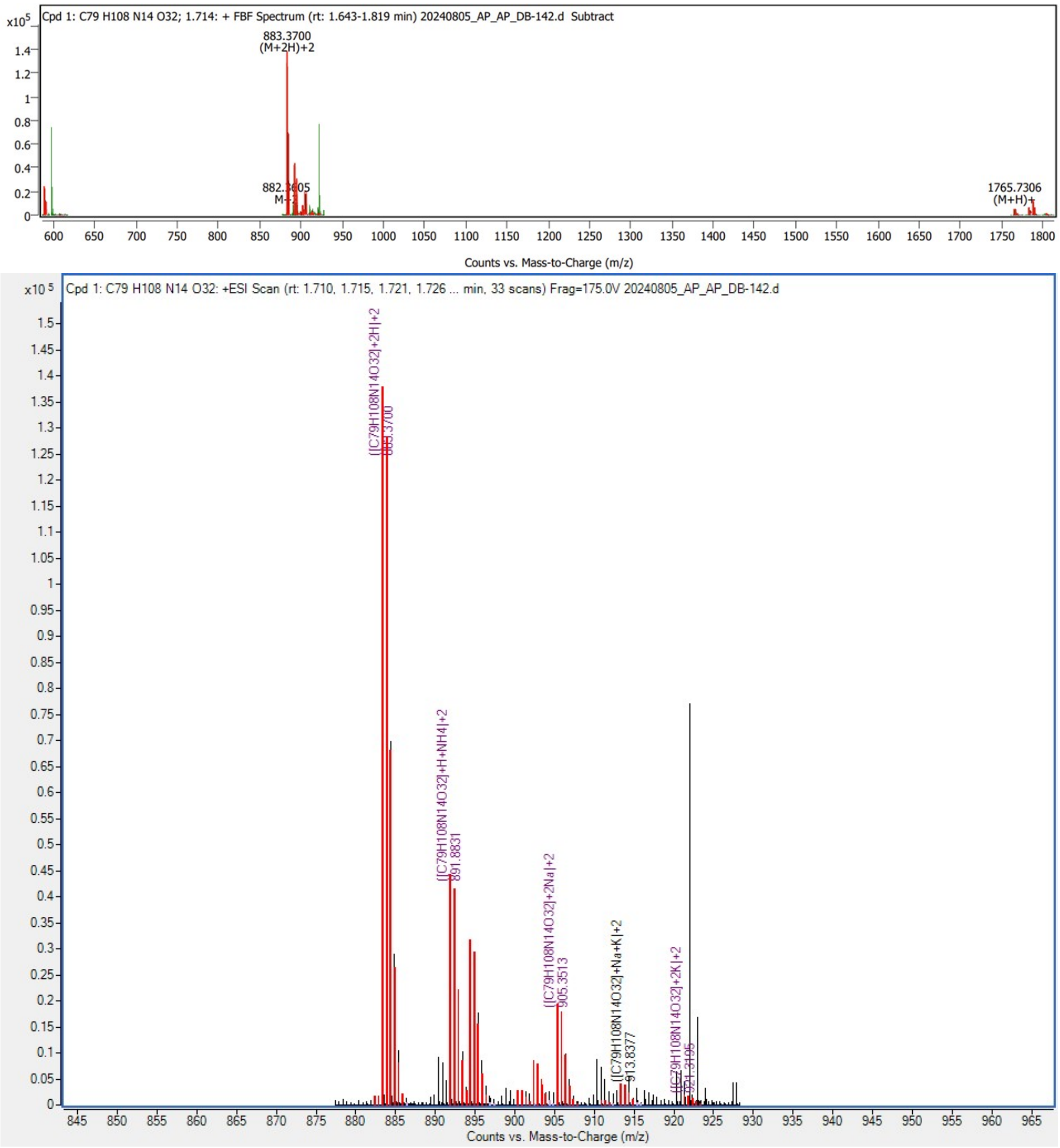

HRMS Spectrum of peptide **1**.

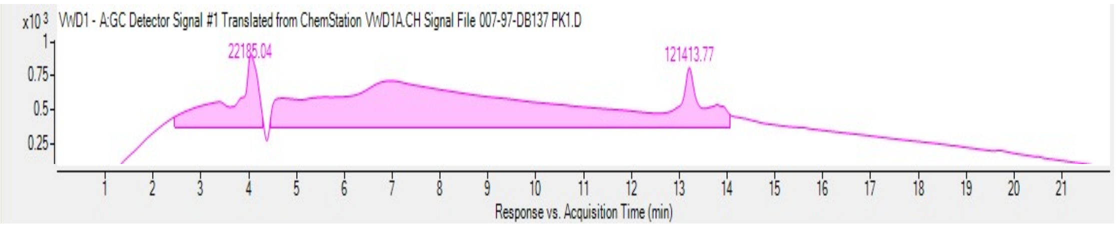

HPLC UV-Vis Spectrum of peptide **2**, rt = 13.241 min, 225 nm.

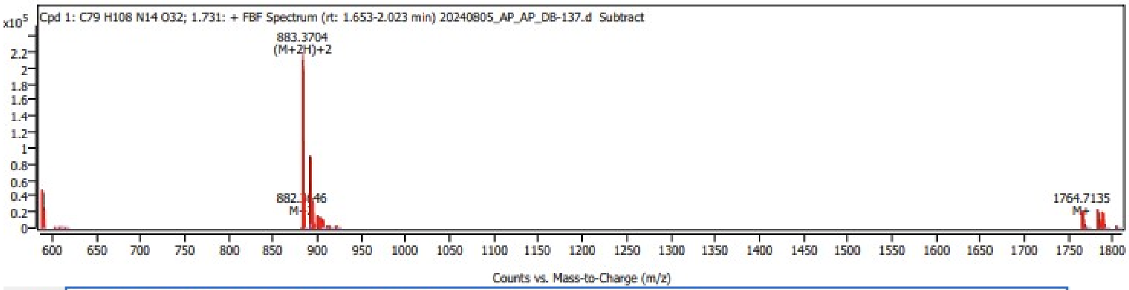

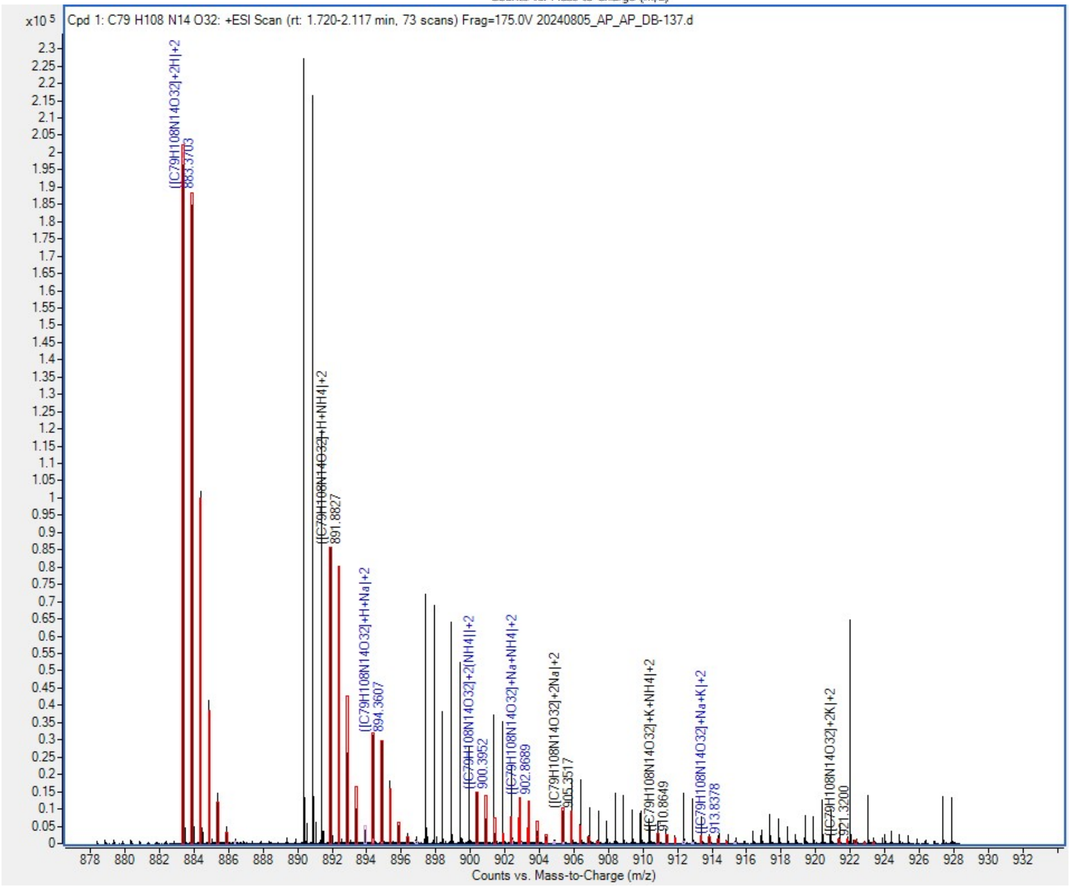

HRMS Spectrum of peptide **1**.

Complexation reactions and nanoparticles characterisation:

Materials: $Zn(NO_3)_2.6H_2O$ was procured from Acros Organics. Strong basic Ion-exchange resin, Purolite® A300 has been received as a generous gift from Purolite Ltd. Triethylamine (TEA) anhydrous, were procured from Fluorochem Ltd (Derbyshire, UK).

Dynamic Light Scattering (DLS) – All solvents used for DLS samples were filtered through 0.2 µm syringe filters. Data for all other samples were collected on a Malvern Pananalytical Zetasizer NanoZS. Data was made up from an accumulation of 3 runs with a minimum of 10 scans per run.

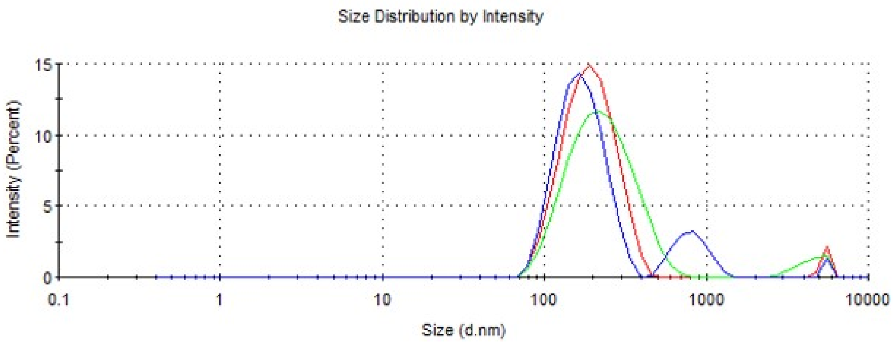

Run	Z-Ave	PdI
**1**	213.7	0.422
**2**	205.5	0.277
**3**	213.7	0.522
**Average of 1-3**	**211.0**	**0.407**

Figure SI1. DLS of **1Znaq**. Measure repeated in triplicate with a minimum of 10 scans per run.

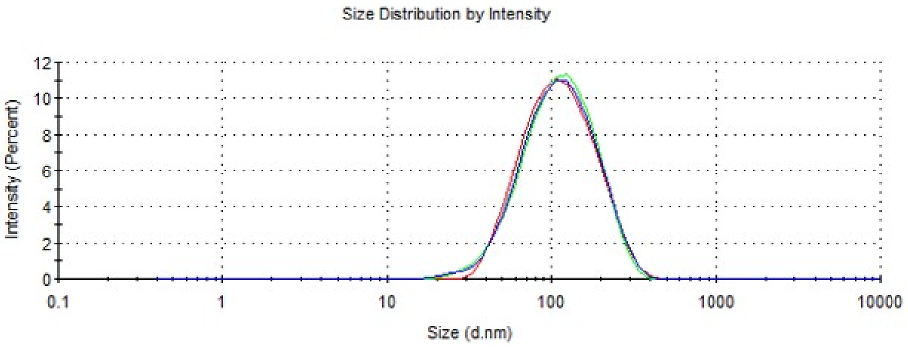

Run	Z-Ave	PdI
**1**	98.29	0.183
**2**	98.36	0.19
**3**	97.92	0.198
**Average of 1-3**	**98.2**	**0.190**

Figure SI2. DLS of **1Zn$_{MeOH}$**. Measure repeated in triplicate with a minimum of 10 scans per run.

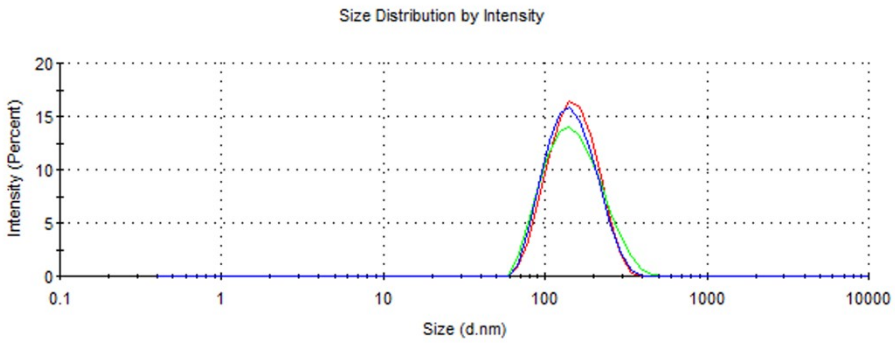

Run	Z-Ave	PdI
**1**	139.3	0.096
**2**	139.7	0.136
**3**	138.6	0.133
**Average of 1-3**	**139.2**	**0.122**

Figure SI3. DLS of **1Zn-Bz**$_{MeOH}$. Measure repeated in triplicate with a minimum of 10 scans per run.

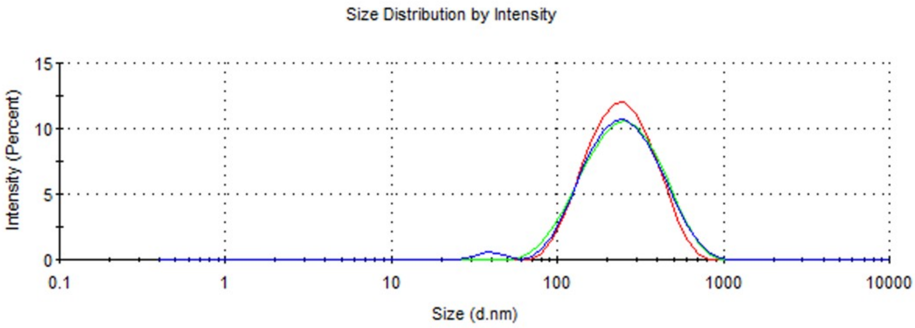

Run	Z-Ave	PdI
**1**	205.3	0.207
**2**	210.2	0.223
**3**	210.7	0.22
**Average of 1-3**	**208.7**	**0.217**

Figure SI4. DLS of **1Zn**$_{EtOH}$. Measure repeated in triplicate with a minimum of 10 scans per run.

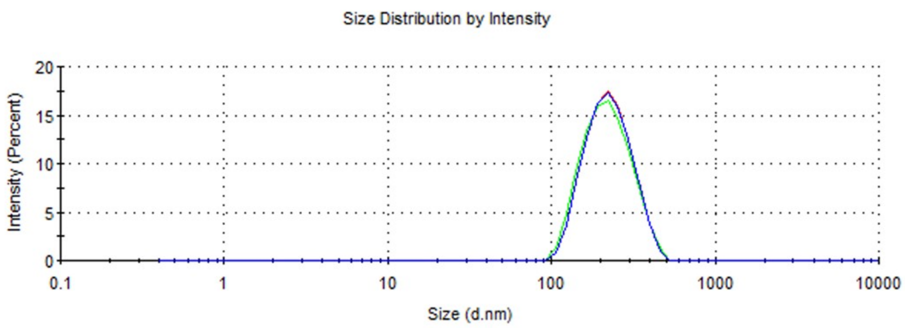

Run	Z-Ave	PdI
**1**	209.1	0.091
**2**	206.5	0.111
**3**	210.3	0.083
**Average of 1-3**	**208.6**	**0.095**

Figure SI5. DLS of **1Zn-Bz**$_{EtOH}$. Measure repeated in triplicate with a minimum of 10 scans per run.

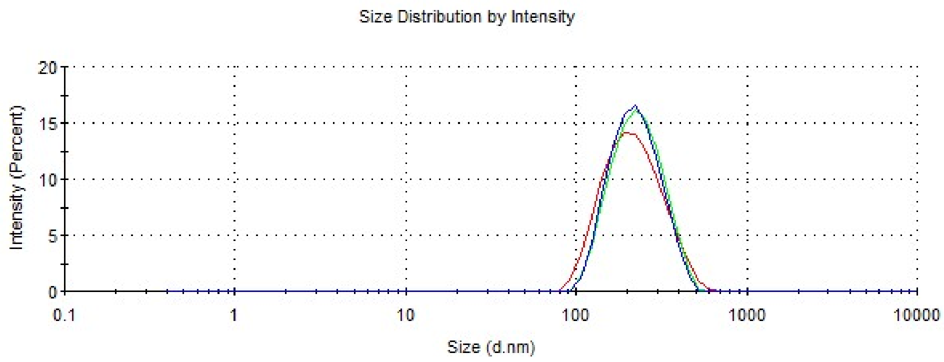

Run	Z-Ave	PdI
**1**	199.5	0.15
**2**	206.7	0.114
**3**	209.7	0.092
**Average of 1-3**	**205.3**	**0.119**

Figure SI6. DLS of **1Zn-Bz**$_{PrOH}$. Measure repeated in triplicate with a minimum of 10 scans per run.

AFM Analysis: 10 µL droplets of each sample (typically at 0.4mg/ml concentration) were deposited on freshly cleaved mica discs (Agar Scientific F7013). For samples with zinc or base the mica was first coated with Ni. A solution of $NiCl_2$ (2 mM) in water (10 µL) was dropcast onto the mica and incubated for 10 mins, excess solution was removed by washing with 1 mL of 0.2-µm syringe-filtered mQ H_2O, and the mica were then dried under a gentle stream of $N_{2\,(g)}$. For aqueous samples after 10-min incubation at room temperature, excess sample was

removed by washing with 1 mL of 0.2-µm syringe-filtered mQ H_2O, and the specimens were then dried under a gentle stream of $N_{2\,(g)}$. For volatile solvents, after 1-min incubation at room temperature excess sample was removed and the mica discs washed with 10 µL (x 3) of 0.2-µm syringe-filtered solvent, and the specimens were then dried under a gentle stream of $N_{2\,(g)}$.Samples were imaged using a Bruker Multimode AFM with a Nanoscope V controller and a ScanAsyst probe (Silicone nitride tip with nominal tip radius = 2 nm, nominal spring constant 0.4 N/m, and nominal resonant frequency 70 kHz). Images were captured at a resolution of 4.88 nm per pixel scanned. All images were processed using Gwyddion analysis software (version 2.65).

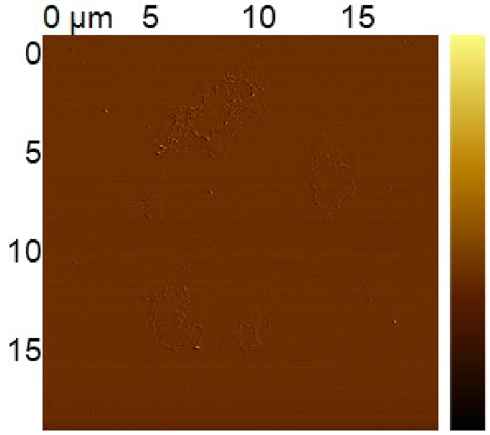

Figure SI7. AFM of **1** in water and triethylamine. No significant morphologies can be identified.

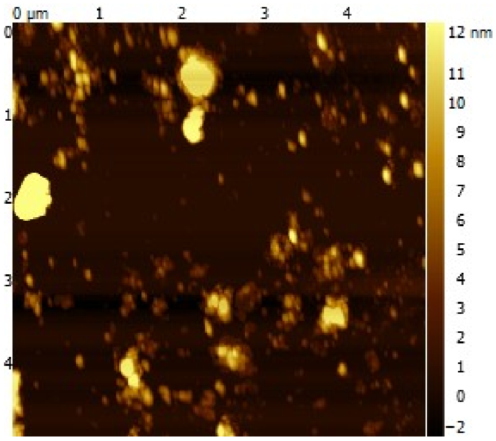

Figure SI8. AFM of **1Zn*aq***

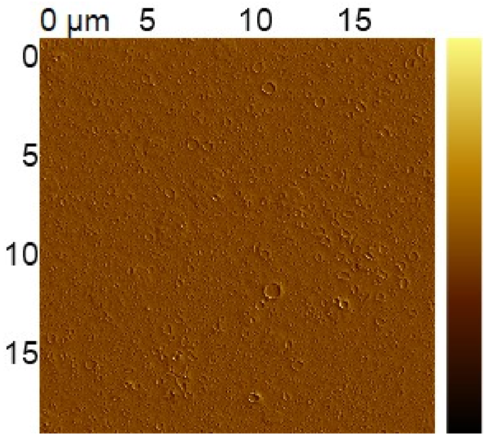

Figure SI9. AFM of **1** in methanol and triethylamine. No significant morphologies can be identified.

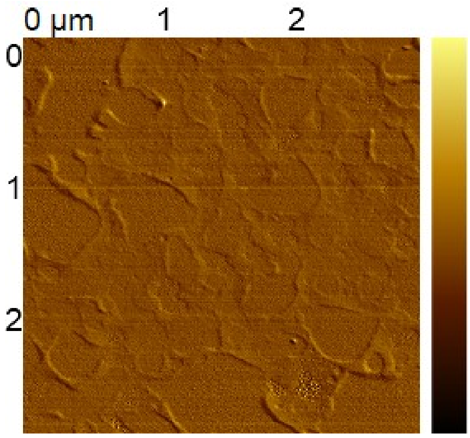

Figure SI10. AFM of **1** in ethanol and triethylamine. No significant morphologies can be identified.

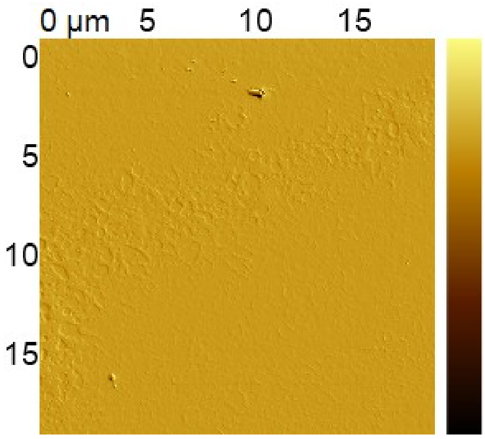

Figure SI11. AFM of **1** in propanol and triethylamine. No significant morphologies can be identified.

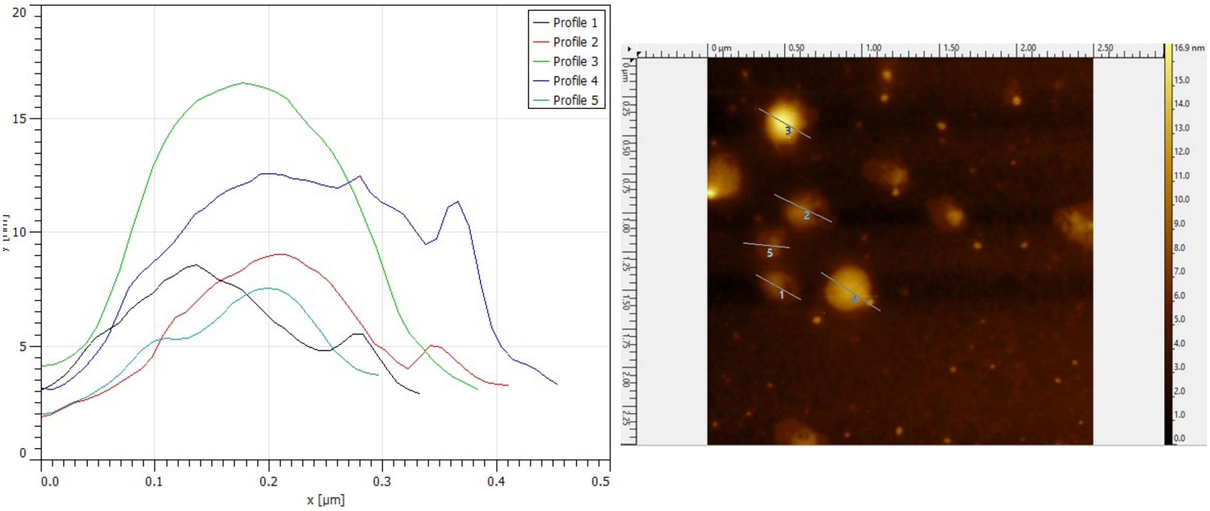

Figure SI12. AFM profile analysis of **1Zn-Bz*_{MeOH}***.

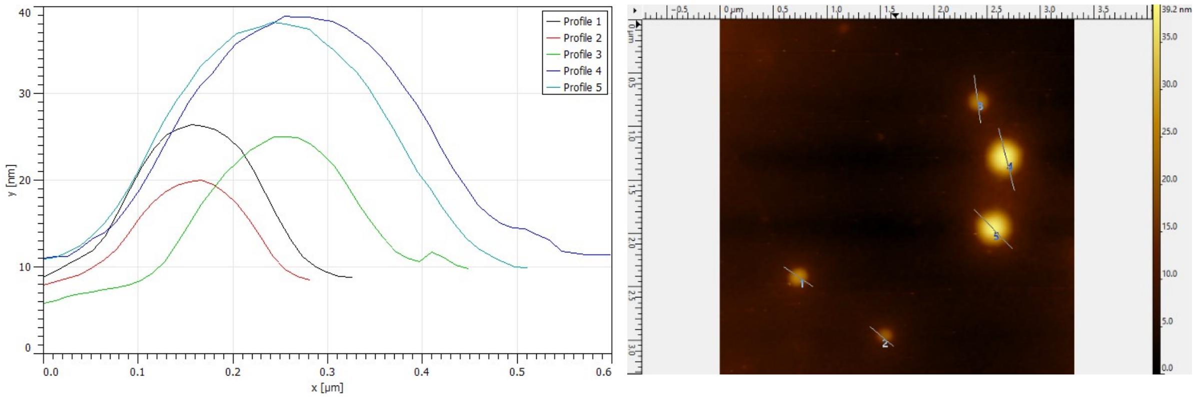

Figure SI13. AFM profile analysis of **1Zn-Bz**.

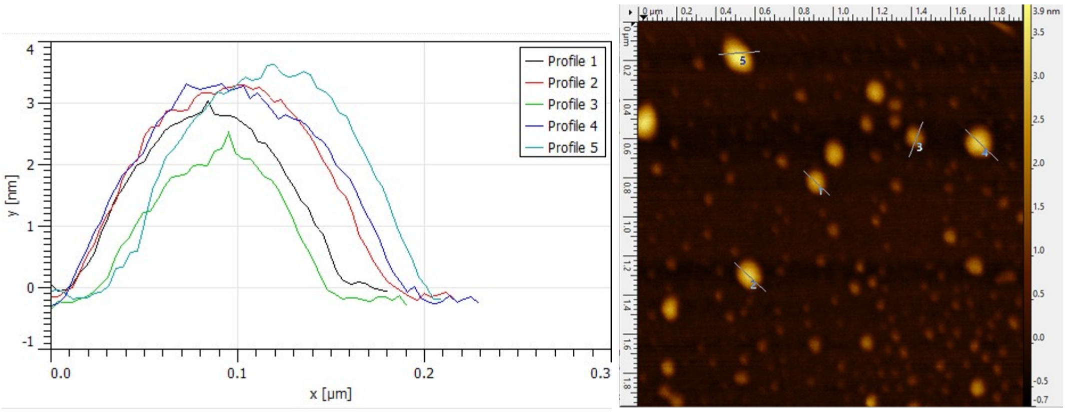

Figure SI14. AFM profile analysis of **1Zn-Bz**.

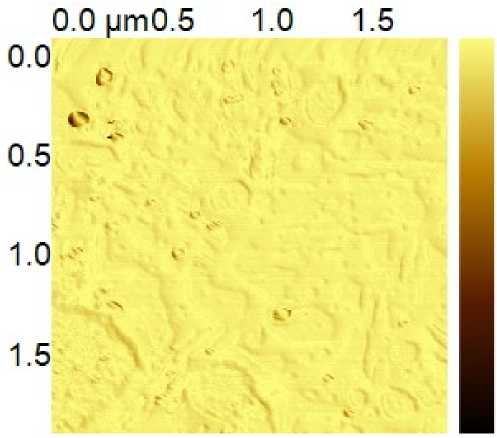

Figure SI15. AFM of **2** in methanol and triethylamine. No significant morphologies can be identified.

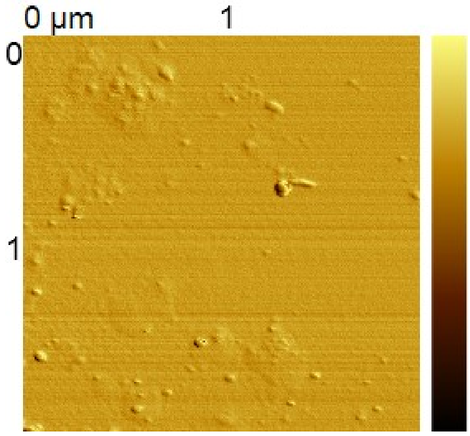

Figure SI16. AFM of **2** in propanol and triethylamine. No significant morphologies can be identified.

TEM Analysis: Samples were prepared by dilution to 0.5 mg/ml and 5 µL droplets were deposited onto 3 mm 400 mesh copper microscope grids covered with holey carbon film (EM Resolutions Ltd) and allowed to settle on the grid for 5 min. Samples were viewed using a JEOL 2100F transmission electron microscope at 100 kV with images recorded on a Gatan Orius CCD camera, or a JEOL 2100Plus at 200kV with TEM images recorded on a Gatan Ultrascan US1000XP CCD camera and dark field scanning TEM (DF-STEM) images recorded using JEOL DF detector.

Peptide **1** and **2** metal complexation reaction:

0.4 mg (1 eq) of peptide (**1** *or* **2**) was dissolved in MeOH/EtOH/PrOH/H_2O (950 µL) in an eppendorf and TEA (6 eq, 1:1 ratio of carboxylic acids to TEA) was added. $Zn(NO_3)_2.6H_2O$ (3 eq, 6:3 ratio of carboxylic acids to Zn^{2+}) in the respective solvent (50 µL) was then added to the solution. The resultant solution was sonicated for 2 mins. 1 equivalent of benzoic acid (6:1 ratio of carboxylic acids to benzoic acid) was added to this solution and sonicated for further 2 mins.

CD Spectroscopy:

CD experiments were carried out on a Jasco J-715 spectropolarimeter. Spectra were recorded using a spectral bandwidth of 190-260 nm, at 20 °C, with a scan rate of 100 nm/min. CD data are given in ellipticity (mdeg). The spectra are formed of 4 accumulations and a spectrum of the solvent blank was subtracted from the raw CD data. A Quartz cell was used with a 1 mm path length using either 125 or 250 μM peptide solutions. All samples were kept in solution for at least 14 days prior to recording CD spectra to ensure the final stable conformation had been achieved due to slow conversion between polyproline helices (i.e. Polyproline II→ Polyproline I). The observed ellipticity has been converted to molar ellipticity (θ) for all spectra, expressed in the units deg.cm^2.dmol^{-1}.

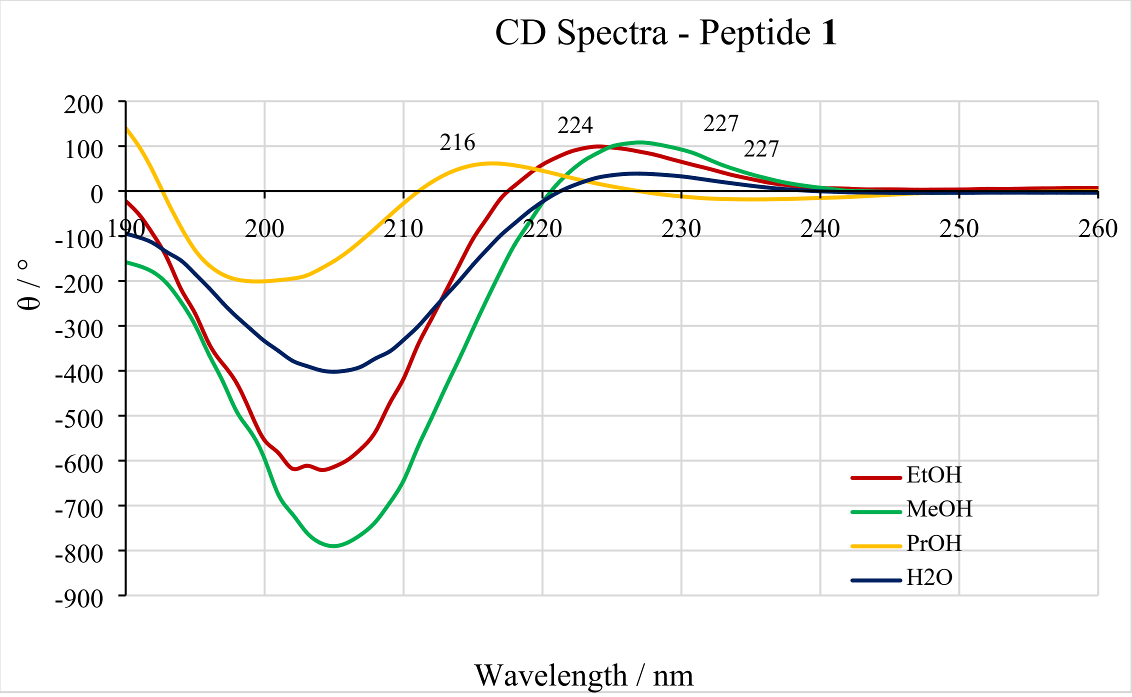

Figure SI17. CD spectra for peptide **1** incubated in MeOH, EtOH, PrOH, and water (125 μM), incubated for 14 days.

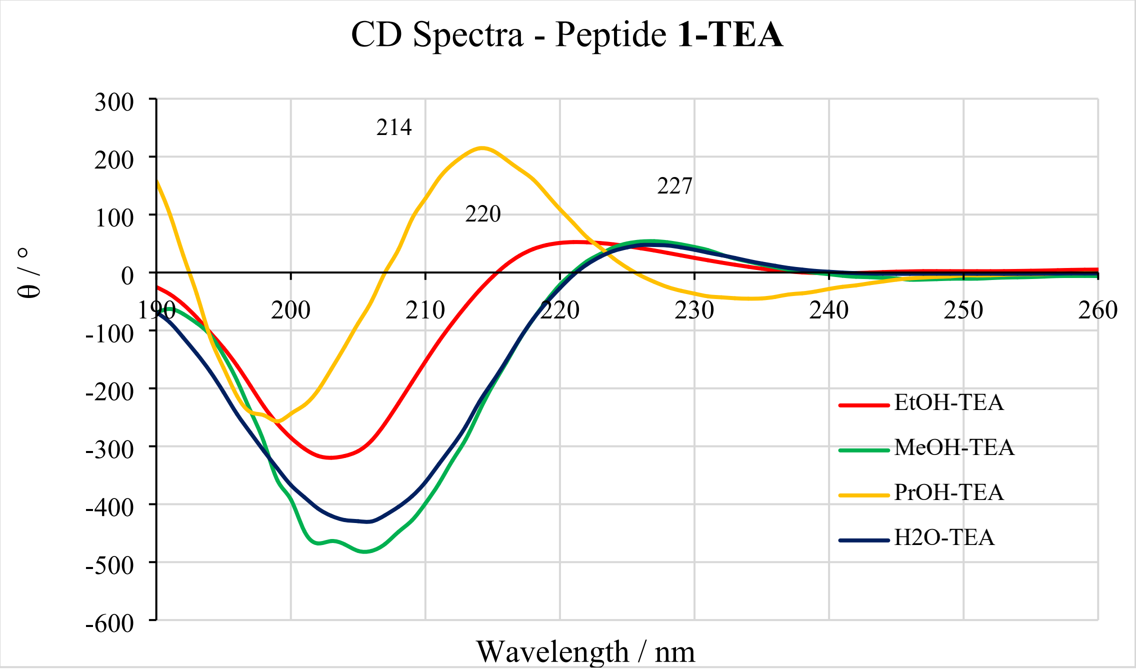

Figure SI18. CD spectra for peptide **1** incubated in MeOH, EtOH, PrOH, and water (125 μM) with triethylamine (6 eq), incubated for 14 days.

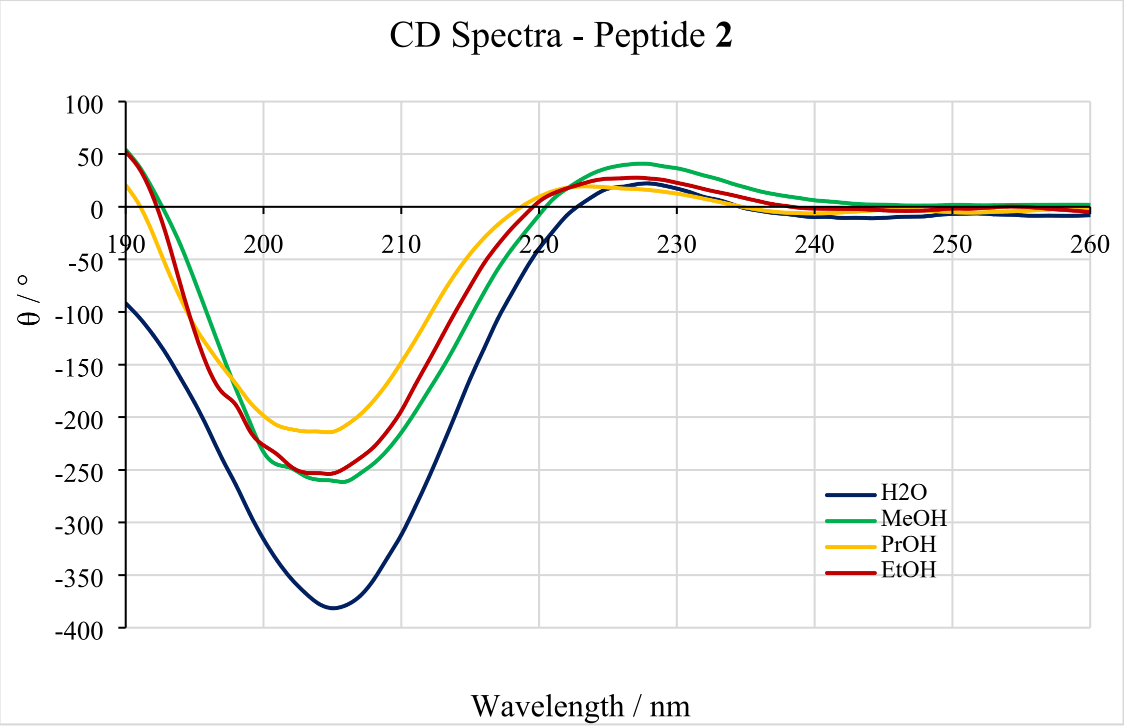

Figure SI19. CD spectra for peptide **2** incubated in MeOH, EtOH, PrOH, and water (125 μM), incubated for 14 days.

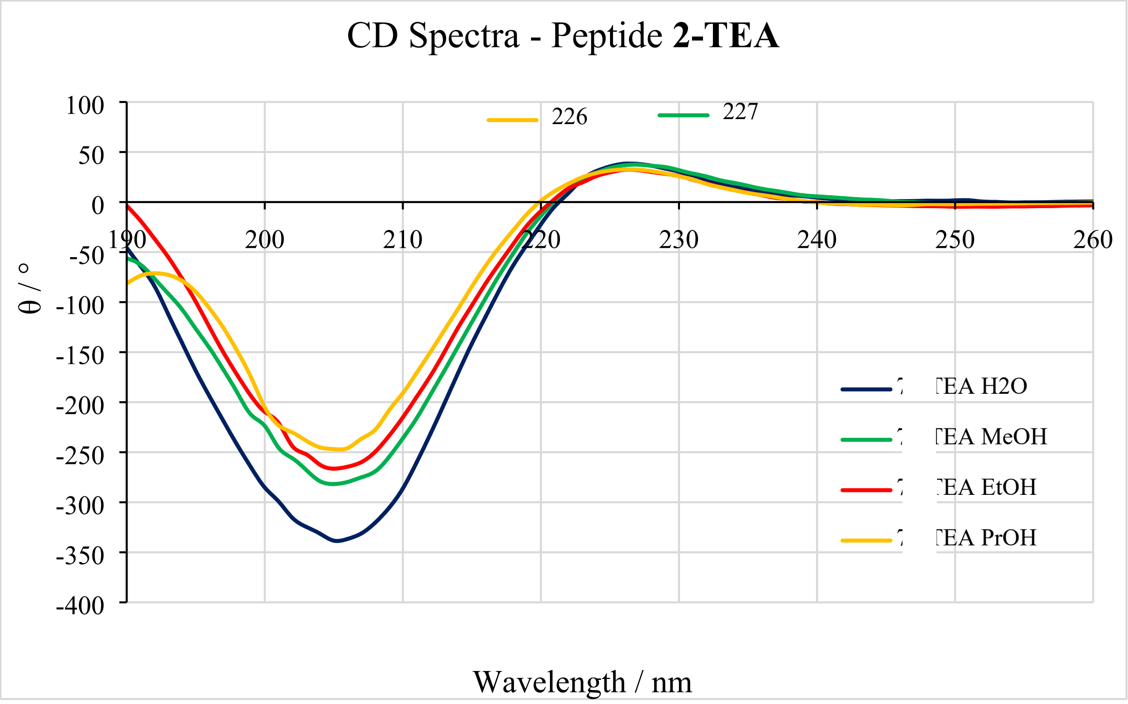

Figure SI20. CD spectra for peptide **2** incubated in MeOH, EtOH, PrOH, and water (125 μM) with triethylamine (6 eq), incubated for 14 days.
